# Expression of young HERV-H loci in the course of colorectal carcinoma and correlation with molecular subtypes

**DOI:** 10.18632/oncotarget.5539

**Published:** 2015-10-23

**Authors:** Philippe Pérot, Christina Susanne Mullins, Magali Naville, Cédric Bressan, Maja Hühns, Michael Gock, Florian Kühn, Jean-Nicolas Volff, Véronique Trillet-Lenoir, Michael Linnebacher, François Mallet

**Affiliations:** ^1^ Cancer Biomarkers Research Group, Joint Unit Hospices Civils de Lyon, bioMérieux, Centre Hospitalier Lyon Sud, Pierre Bénite, France; ^2^ Centre d'Investigation des Thérapeutiques en Oncologie et Hématologie, EMR 3738 Lyon Claude Bernard University, Institut de Cancérologie des Hospices Civils de Lyon, France; ^3^ Institut de Génomique Fonctionnelle de Lyon, Ecole Normale Supérieure de Lyon, CNRS/Université Lyon I, Lyon, France; ^4^ Institute of Pathology, University Medicine Rostock, Rostock, Germany; ^5^ Department of General, Thoracic, Vascular and Transplantation Surgery, University Medicine Rostock, Rostock, Germany; ^6^ Department of General Surgery, Molecular Oncology and Immunotherapy, University Medicine Rostock, Rostock, Germany; ^7^ Current address: Institut Pasteur, Laboratory for Pathogen Discovery, Paris, France

**Keywords:** colorectal cancer, HERV-H, microsatellite instability, qRT-PCR, biomarker

## Abstract

**Background:**

Expression of the human endogenous retrovirus (HERV)-H family has been associated with colorectal carcinomas (CRC), yet no individual HERV-H locus expression has been thoroughly correlated with clinical data.

Here, we characterized HERV-H reactivations in clinical CRC samples by integrating expression profiles, molecular patterns and clinical data. Expression of relevant HERV-H sequences was analyzed by qRT-PCR on two well-defined clinical cohorts (*n* = 139 pairs of tumor and adjacent normal colon tissue) including samples from adenomas (*n* = 21) and liver metastases (*n* = 16). Correlations with clinical and molecular data were assessed.

**Results:**

CRC specific HERV-H sequences were validated and found expressed throughout CRC disease progression. Correlations between HERV-H expression and lymph node invasion of tumor cells (*p* = 0.0006) as well as microsatellite instable tumors (*p* < 0.0001) were established. No association with regard to age, tumor localization, grading or common mutations became apparent. Interestingly, CRC expressed elements belonged to specific young HERV-H subfamilies and their 5′ LTR often presented active histone marks.

**Conclusion:**

These results suggest a functional role of HERV-H sequences in colorectal carcinogenesis. The pronounced connection with microsatellite instability warrants a more detailed investigation. Thus, HERV-H sequences in addition to tumor specific mutations may represent clinically relevant, truly CRC specific markers for diagnostic, prognostic and therapeutic purposes.

## INTRODUCTION

### Endogenous retroviruses

Endogenous retroviruses are an inherent part of mammalian genomes and typically consist of viral gag, pol and env sequences flanked by long terminal repeats (LTR). This genomic heritage originates from ancestral and independent retroviral infections within the germ line. Complex reinfection, retro-transposition, propagation and error-prone steps occurred during evolution, leading to the formation of multi-copy sequences arranged in families [1]. In humans, about 100 human endogenous retrovirus (HERV) families are identified [2], yet only about thirty have been reasonably well studied [3]. Each family contains tens to thousands of distinct loci scattered throughout the human genome, representing an overall pool of approximately 200,000 individual HERV loci. Together with the mammalian apparent LTR-retrotransposons, LTR retroelements span roughly 8% of the chromatin [2]. To date, all characterized HERV elements are replication defective. It is generally accepted that HERV are silent due to mutations and epigenetic regulation [4]. Nevertheless, in some contexts, HERV have become *bona fide* genes that contribute to biological functions. For instance, the Syncytin-1 envelope glycoprotein is essential for human placentation [5, 6]. However, the major contribution of (H)ERV sequences to the evolution of species and functional genomics relies presumably on their LTR. They can trigger chromosomal breaks through recombination events [7] and serve as natural or alternative promoters and enhancers capable of modulating transcription [8].

### HERV-H and colorectal cancer

Colorectal cancer (CRC) remains the second cause of cancer-related deaths in Europe and in the United States and its incidence increases in developing countries. The diagnosis of CRC depends primarily on colonoscopy. Some molecular markers are in clinical use, e.g. the dosage of the carcinoembryonic antigen in serum [9], but no marker indicates the early conversion of adenomatous polyps to adenocarcinoma. There is therefore a demand for (early) diagnostic markers, ideally based on non-invasive sampling methods. In addition, CRC is closely connected to genetic background (e.g. familial adenomatous polyposis and hereditary non-polyposis colorectal cancer (HNPCC) or more broadly called Lynch syndrome), chronic inflammation, lifestyle and dietary habits [10]. At least three molecular subtypes of CRC are currently well recognized: (I) the chromosomal instable (CIN) tumors (characterized by aneuploidy), (II) the microsatellite instable (MSI) tumors (loss of the DNA mismatch repair system causes mutations especially in repetitive DNA sequences) and (III) the tumors presenting with the CpG island methylation phenotype with frequent inactivation of tumor-suppressor regions by methylation [11].

A major consequence of the abundance of LTR regulatory elements within the human genome is that permissive HERV reactivations are often associated with pathological contexts including cancer. Transcripts from HERV-K HML-2 have been associated with numerous cancers including melanoma [12], leukemia and lymphoma [13] as well as tumors of the breast [14, 15], testis [15] and ovary [16]. The HERV-E family has been associated with prostate, kidney, ovarian and uterine cancers [17, 18]. Conversely, the expression of the HERV-H family has been previously associated essentially with CRC [15, 19], but, to date, the identification of individual reactivated HERV-H loci remains poor. One unique HERV-H locus on Xp22.3 has been repeatedly described to be up-regulated in CRC [22, 23].

### Recent findings and purpose of the study

We recently used a dedicated Affymetrix custom microarray to gain insights into the HERV transcriptome using a composite panel of 40 normal and 39 tumor RNA samples, including breast, colon, lung, ovary, prostate, testis, uterus, and placenta samples. This led to the identification of 284 differentially expressed HERV loci including 166 HERV-H elements in paired colon tissues (*n* = 4 pairs of tumor and adjacent normal tissue). Using partitioning clustering, a restricted list of 21 HERV-H loci was identified. Although their expression appeared specific to CRC, it relied only on a limited number of samples [24]. Following these results, we herein sought to deeply characterize HERV-H reactivations in CRC by integrating expression profiles with molecular and clinical data for a large cohort. HERV-H locus-specific qRT-PCR systems (*n* = 19) were designed and validated using a small sample series (*n* = 32 tumors and *n* = 21 corresponding normal tissues). After a short list of five HERV-H candidate sequences has been circumscribed, their expression was analyzed in two well-defined and independent clinical cohorts composed of tumor and normal adjacent colon tissues (*n* = 139 pairs). Additionally, samples from early and late stages of the disease (i.e. adenomas (*n* = 21) and metastases (*n* = 16)) were analyzed. Finally, associations of HERV-H expression with clinical and molecular parameters were investigated.

## RESULTS

### Conception of HERV-H locus-specific qRT-PCR systems and selection of HERV-H candidates

HERV-H locus-specific qRT-PCR systems (*n* = 19) were meticulously designed and validated to secure locus specificness (Supplementary Figure S1). These systems were applied to a small series of tumor and normal samples (*n* = 32 tumors and *n* = 21 corresponding normal tissues from commercial sources) for subsequent transfer to clinically relevant samples and as proof of concept for these HERV-H loci. Generally, no expression (normalized mean expression, as defined in Material and Methods, was 13 and highest expression was 110) for any of the selected HERV-H sequences was observed in normal tissue. The expression in the cancer specimen ranged from no expression to peak levels of 546 (Supplementary Figure S2). Controls, as determined by a previous study [24], were added to ensure accurate interpretation of results: demonstrating (I) colonic origin – the HERV-H positive control was solely expressed in colon tissue (both normal and tumorous), whereas the expression of the HERV-H negative control was restricted to testicular cancer (positive control: 1900007_h_L5U3 and negative control: 1900006_h_env) and (II) tumorous origin (matrix metalloproteinase (MMP7) and osteopontin (OPN)) of the tissue. Of note, when distinguishing samples from Asian and Caucasian populations, no differences were observed (Supplementary Figure S3).

### Multi-centric study of HERV-H expression in clinical CRC samples

**Figure 1:**
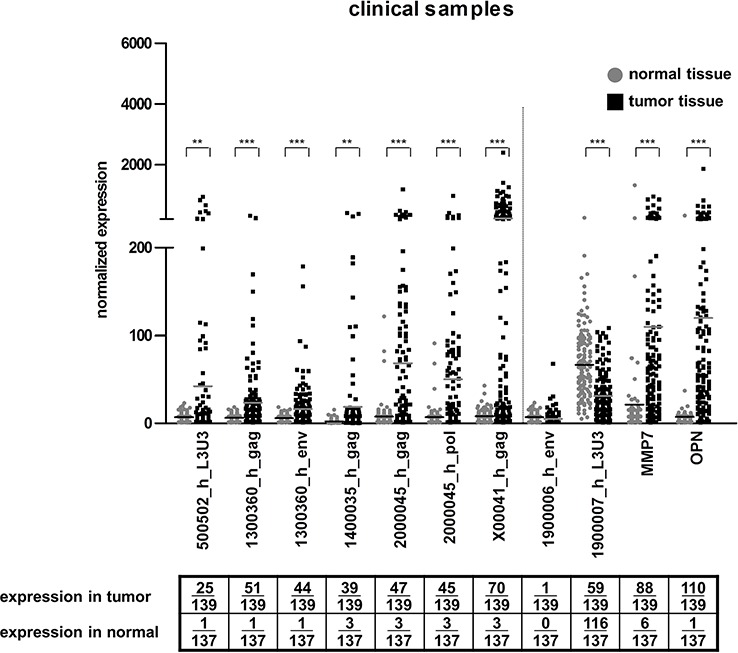
HERV-H expression in clinical samples The expression of five HERV-H sequences (represented by eight qRT-PCR systems) for CRC (black squares) and corresponding normal (grey dots) tissue is depicted in the dot plot. Statistically significant differences in expression between normal and tumorous tissue are indicated by stars (*p < 0.05, **p < 0.01, ***p < 0.001, *t*-test). For better interpretation of the results, controls for tissue specificity (1900006_h_env = negative control, 1900007_h_L3U3 = positive control) and cancerous origin (MMP7 and OPN) were added and are separated from the HERV-H sequences by the dotted line. Absolute numbers of samples with expression (= values greater than mean expression value in normal tissue plus three times standard deviation was calculated individually for each PCR system) for tumorous and normal tissue are given in the table below.

Based on the results obtained with the commercial samples, a shorter list of HERV-H candidate sequences (5 loci, 7 regions; Supplementary Figure S4) was circumscribed. Further qRT-PCR experiments were performed over two well-defined and independent clinical cohorts composed of tumor and normal adjacent colon tissues (*n* = 139 CRC tumors and 137 corresponding adjacent normal tissue in total) (Figure 1). Again, virtually no expression (6/137) in normal colon tissue was observed. Varying degrees of expression could be detected in tumors for all systems analyzed. HERV-H sequences were expressed in 100 out of the 139 CRC samples. Thirty samples were positive for a single locus whereas 70 samples expressed two to five HERV-H loci. The previously described sequence on chromosome X (X00041_h_gag) had the highest expression frequency with detection in half of the tumors, followed by a third of tumors expressing a locus on chromosome 20 (2000045_h). Other significant expressions, ranging from 17% to 27%, were detected for the loci 500502_h, 1400035_h and 1300360_h. The positive and negative controls defined within the HERV-H family exhibited an opposite behavior. There was only one false positive out of the 276 tissue samples (for the negative control) but 175 rightfully positive (for the positive control). Conventional tumor markers MMP7 and OPN were found expressed in 63% and 79% of the tumors and in 4% and 1% of the normal tissues, respectively. When discerning samples collected at the biobanks in Rostock and Reims, no differences in the trends became apparent (Supplementary Figure S5).

### HERV-H reactivations correlate with MSI and lymph node status

**Figure 2:**
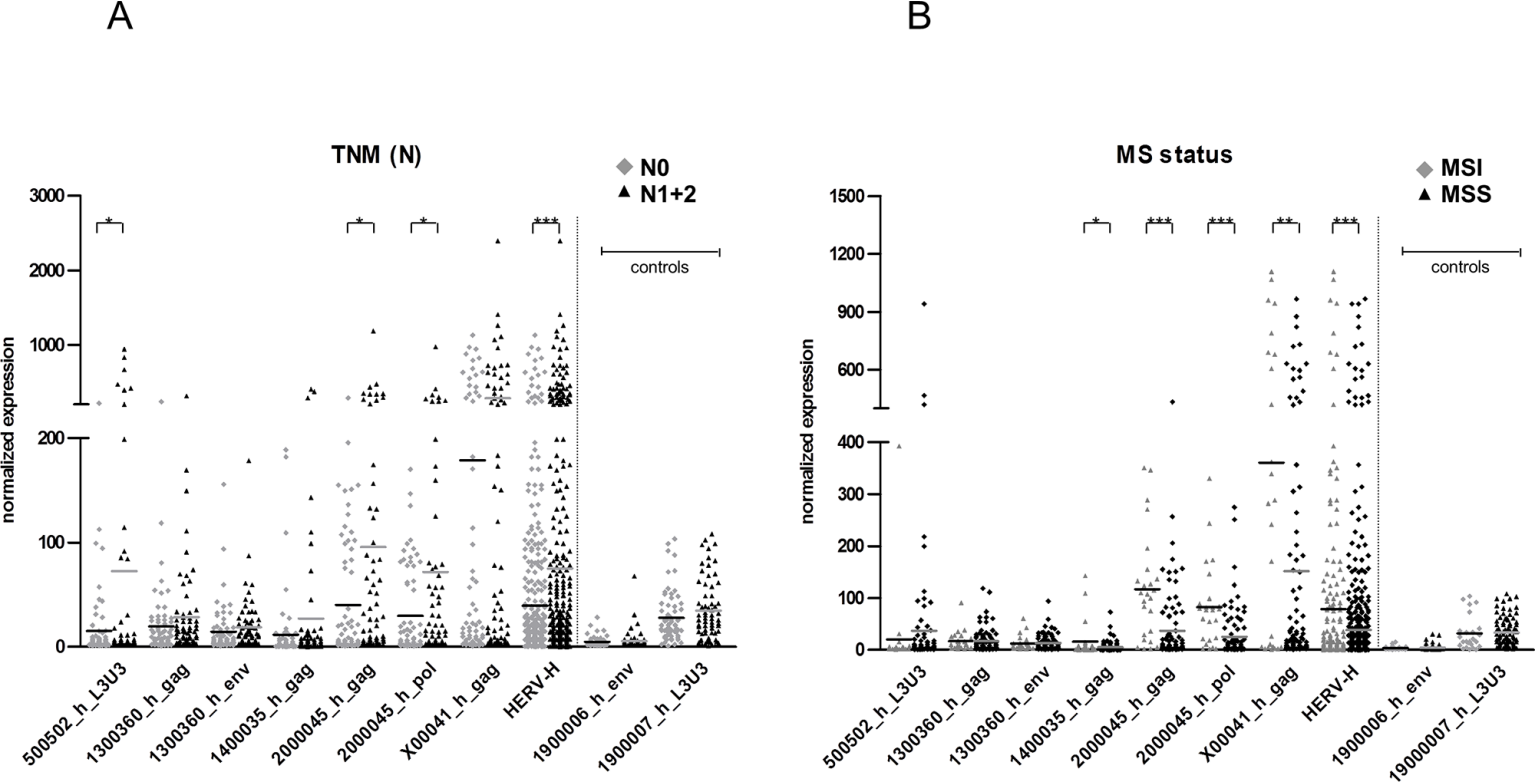
Correlation of HERV-H expression with tumor cell infiltration into lymph nodes and MS status The expression of five HERV-H sequences for **A.** tumors with infiltration of tumor cells into lymph nodes (black triangles) or not (grey diamonds) and **B.** MSI (grey diamonds) and MSS (black triangles) tumors is depicted in the dot plot. Statistically significant differences in expression between MSI and MSS tumors are indicated by stars (*p < 0.05, **p < 0.01, ***p < 0.001, *t*-test). HERV-H: combination of all eight sequences, N0: no tumor cells in regional lymph nodes, N1: infiltration of tumor cells into regional lymph nodes, N2: infiltration of tumor cells into more distant lymph nodes; MSI: microsatellite instability, MSS: microsatellite stability. The expression of the two colon control sequences belonging to the same HERV family (HERV-H) is depicted to demonstrate no difference between the statuses.

Possible connections between HERV-H expression and molecular as well as clinical parameters were assessed (Table 1 and Figure 2A and 2B). No association with regard to age, tumor localization, grading or common mutations (APC, TP53, KRAS and BRAF) became apparent. However, the presence of tumor cells in lymph nodes (N status) correlated with HERV-H expression (*p* = 0.0006) and moreover, HERV-H expression was significantly higher in MSI positive tumors (*p* < 0.0001). More precisely, the loci on chromosomes 5 and 20 show a characteristic behavior for the association with nodal tumor cell infiltration whereas for the association with MSI, the loci on chromosomes 20, X and 14 are most representative. Conversely, the locus on chromosome 5 (500502_h) has an opposite (statistically not significant) behavior, where expression tends to be higher in MSS than MSI samples.

**Table 1:** Clinical and molecular information for CRC patients

		*Commercial samples*	*Clinical samples*
*gender*	*male*	20 (65%)	73 (53%)
	*female*	11 (35%)	66 (47%)
*age*	*average*	66 years	71 years
	*range*	37–89 years	21–90 years
*mutations*	*APC*	n.a.	6/17 (35%)
	*TP53*	n.a.	7/17 (41%)
	*KRAS*	n.a.	27/79 (34%)
	*BRAF*	n.a.	10/79 (13%)
*MS status*	*MSS*	n.a.	91 (78%)
	*MSI*	n.a.	25 (22%)

The table summarizes the information on patients’ gender, age, common mutations (TP53, APC, KRAS, BRAF) and microsatellite (MS) status of the tumors (MSS: microsatellite stable; MSI: microsatellite instable) for samples obtained from commercial suppliers (*n* = 32 tumor and *n* = 21 matching normal tissue) and biobanks (*n* = 139 pairs of tumor and adjacent normal colon tissue); n.a. = not assessed.

The fact that the distribution of HERV-H loci expression ranges from none, a single one to all loci analyzed, underlines the expression heterogeneity of HERV-H.

### HERV-H reactivations in three synchronal tumors

**Figure 3:**
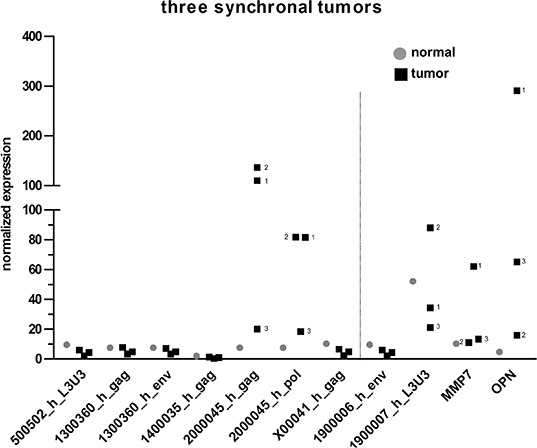
Comparison of HERV-H expression in three synchronal tumors The expression of five HERV-H sequences for three synchronal tumors (black squares) and corresponding normal (grey dots) tissue of an HNPCC/Lynch patient is depicted in the dot plot. In case of differential expression of the three tumors, numbers corresponding to their localization from proximal to distal are added (1: descending colon, 2: sigmoid colon, 3: rectum). For better interpretation of the results, controls for tissue specificity (1900006_h_env = negative control, 1900007_h_L3U3 = positive control) and cancerous origin (MMP7 and OPN) were added and are separated from the HERV-H sequences by the dotted line.

Accordingly, three synchronal, MSI positive tumors of a 45 year old male HNPCC/Lynch patient who underwent subtotal colectomy were analyzed for expression of the five selected HERV-H sequences. All tumors were adenocarcinomas, highest tumor stage according to the UICC classification was IIb (G3 pT4b pN0 (0/74) L0 V1 R0 cM0). Tumor 1 was localized in the descending colon, tumor 2 in the sigmoid colon and tumor 3 in the rectum. Two out of the three tumors (tumor 1 and 2, both from the colon) exclusively expressed the locus on chromosome 20 (2000045_h) whereas the rectal tumor (number 3) did not express HERV-H sequences, or only at very low levels of 2000045_h (Figure 3). The expression of solely one HERV-H locus (as described for the three synchronal tumors here) is not a unique phenomenon, since in total 30 CRC samples express a single locus (see above); with both loci on chromosomes 20 and X expressed uniquely in nine CRC samples each. Conversely, the tumor markers MMP7 and OPN were only expressed in tumor 1 (colon) and tumors 1 (colon) and 3 (rectum), respectively.

According to these data, HERV-H expression does not seem to be linked to a genetic predisposition, but rather a unique acquisition during CRC tumor formation.

### HERV-H expression during CRC disease progression

**Figure 4:**
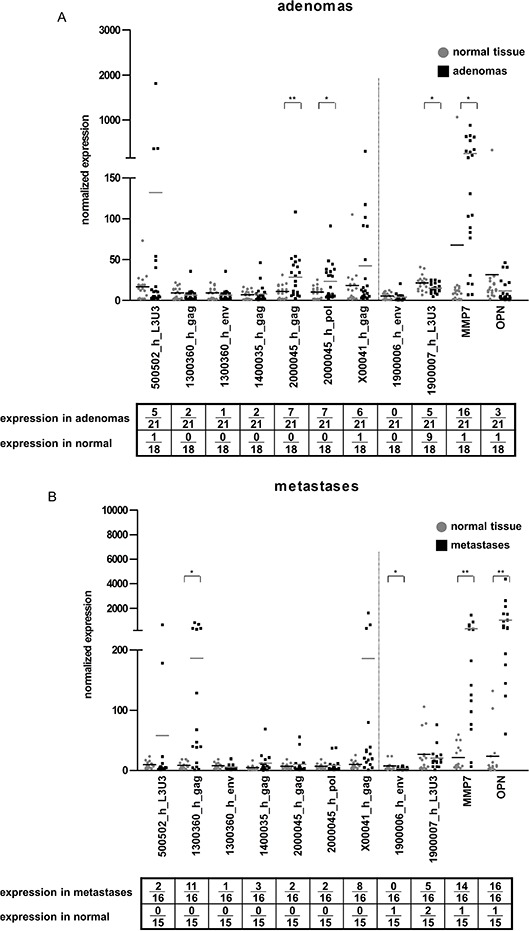

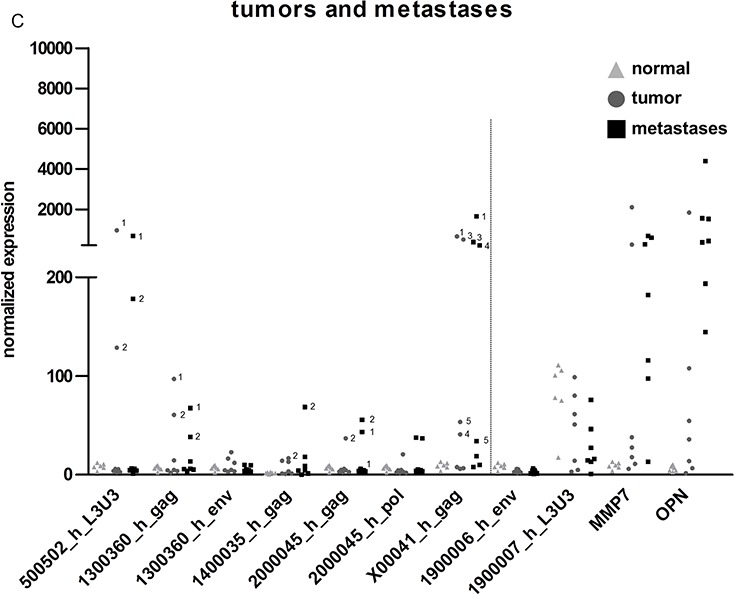
HERV-H expression in adenomas, metastases and comparison of matched tumor and metastasis pairs The expression of five HERV-H sequences from **A.** adenomas and **B.** metastases comparing the pathological sample (black squares) and corresponding normal (grey dots) tissue is depicted in the dot plot. **C.** direct comparison of tumors (dark grey dot) and corresponding CRC liver metastases (black square) and normal tissue (light grey triangle) of seven patients. For better interpretation of the results, controls for tissue specificity (1900006_h_env = negative control, 1900007_h_L3U3 = positive control) and cancerous origin (MMP7 and OPN) were added and are separated from the HERV-H sequences by the dotted line.

Continuing the investigation if HERV-H expression is linked to tumorigenesis, additional samples from adenomas (*n* = 21) and liver metastases (*n* = 16) were analyzed to help represent the complete natural course of the disease. HERV-H expression was already present in adenomas. A third of the adenomas expressed the locus on chromosome 20, followed by 29% of the samples being positive for the sequence on the X chromosome and 24% for the locus on chromosome 5 (Figure 4A). In metastases, HERV-H expression tended to be lower than in the tumor samples, however, 69% of metastases expressed the locus on chromosome 13 (1300360_h_gag) and half the HERV-H sequence on the X chromosome (Figure 4B). Only in one metastasis very low expression of the env gene of 1300360_h was detected. This strong discrepancy in the expression of two genes from the same locus is merely found for the env gene of chromosome 13 in metastases. In adenomas, only one sample solely expresses the gag gene of chromosome 1300360_h and one sample both genes. The gag gene alone is expressed in twelve CRC tumor cases, the env gene alone is present in three samples and 40 CRC express both genes at the same time. Thus, the isolated expression of 1300360_h_gag in metastases could either be the result of alternative splicing or genomic loss of the env region in the metastatic process. Direct comparison of tumors and metastases from the same patient (*n* = 7 pairs) revealed no general differences in expression profiles. With regard to the sequence on chromosome 13, no conclusions can be drawn since none of the corresponding tumors expressed the gag gene (Figure 4C).

Overall, HERV-H expression was found to be stable throughout CRC disease progression.

### HERV-H expression in liver tissue and organs with common sites of metastasis

**Figure 5:**
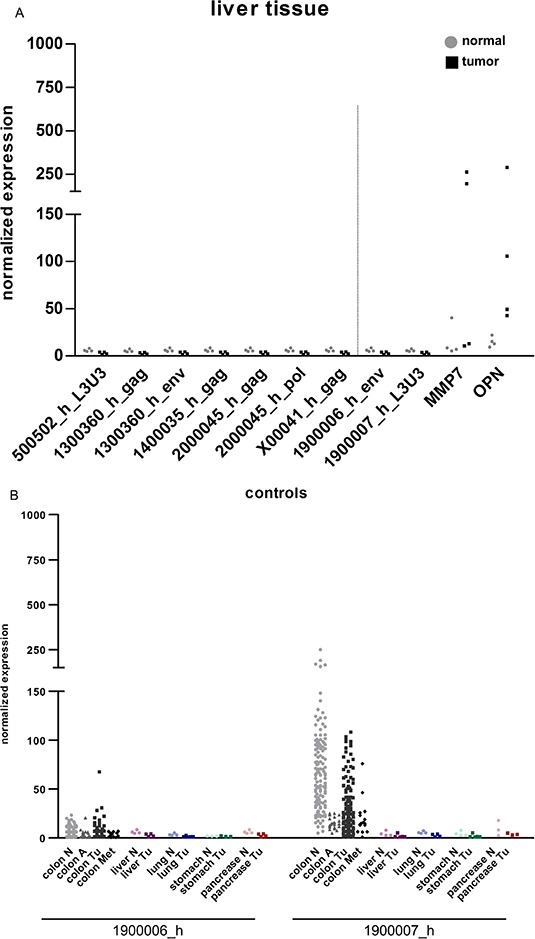
HERV-H expression in samples of organs with common sites of metastasis with CRC The expression of five HERV-H sequences in **A.** liver tumors and normal liver tissue was analyzed. For comparison of the results with colon samples controls for tissue specificity (1900006_h_env = negative control, 1900007_h_L3U3 = positive control) and cancerous origin (MMP7 and OPN) were added and are separated from the HERV-H sequences by the dotted line. **B.** The expression of the HERV-H negative (1900006_h) and positive (1900007_h) control was analyzed in samples of the colon (normal tissue, adenomas, tumors and liver metastases), liver (normal and tumorous tissue), lung (normal and tumorous tissue), stomach (normal and tumorous tissue) and pancreas (normal and tumorous tissue).

Samples from liver tumors and adjacent normal tissue (*n* = 4), were analyzed for the expression of the allegedly CRC specific HERV-H loci. None of the described CRC specific HERV-H loci were expressed in either tumor or normal liver tissue. In contrast, the tumor controls MMP7 and OPN were clearly expressed in the cancerous liver samples (Figure 5A). Further paired tumorous and adjacent normal tissue having common sites of metastasis with CRC (lung (*n* = 5), stomach (*n* = 3) and pancreas (*n* = 3)) were analyzed [41] to verify CRC restricted expression of the selected HERV-H sequences. Expression of the two HERV-H controls was analyzed to investigate the potential for predicting the organ of origin in metastases, i.e. colon in liver metastases. No sample, other than those with colonic origin, expressed the HERV-H positive control 1900006_h; whereas the HERV-H negative control was absent in all samples (Figure 5B). Further, no expression of any HERV-H locus was observed for samples derived from lung normal/tumor tissue pairs. MMP7 and OPN were always (highly) expressed in these tumors samples, even being expressed in some normal counterparts. Regarding gastric samples, low expression of the HERV-H sequence on chromosome 20 was detectable in normal tissues but was always absent in the tumorous counterparts. Conversely, MMP7 and OPN expression tended to be associated with the tumor tissue. In all samples of pancreatic tissue (tumor and normal) expression of 2000045_h was detected; expression appeared increased in pancreatic tumors. Additionally, one pancreatic tumor showed low level reactivation of the sequence on chromosome 5 (Supplementary Figure S6).

### Evolutionary and functional characterization of the CRC specific HERV-H loci in comparison to gonads specific, constitutively active and silent HERV-H sequences

**Figure 6:**
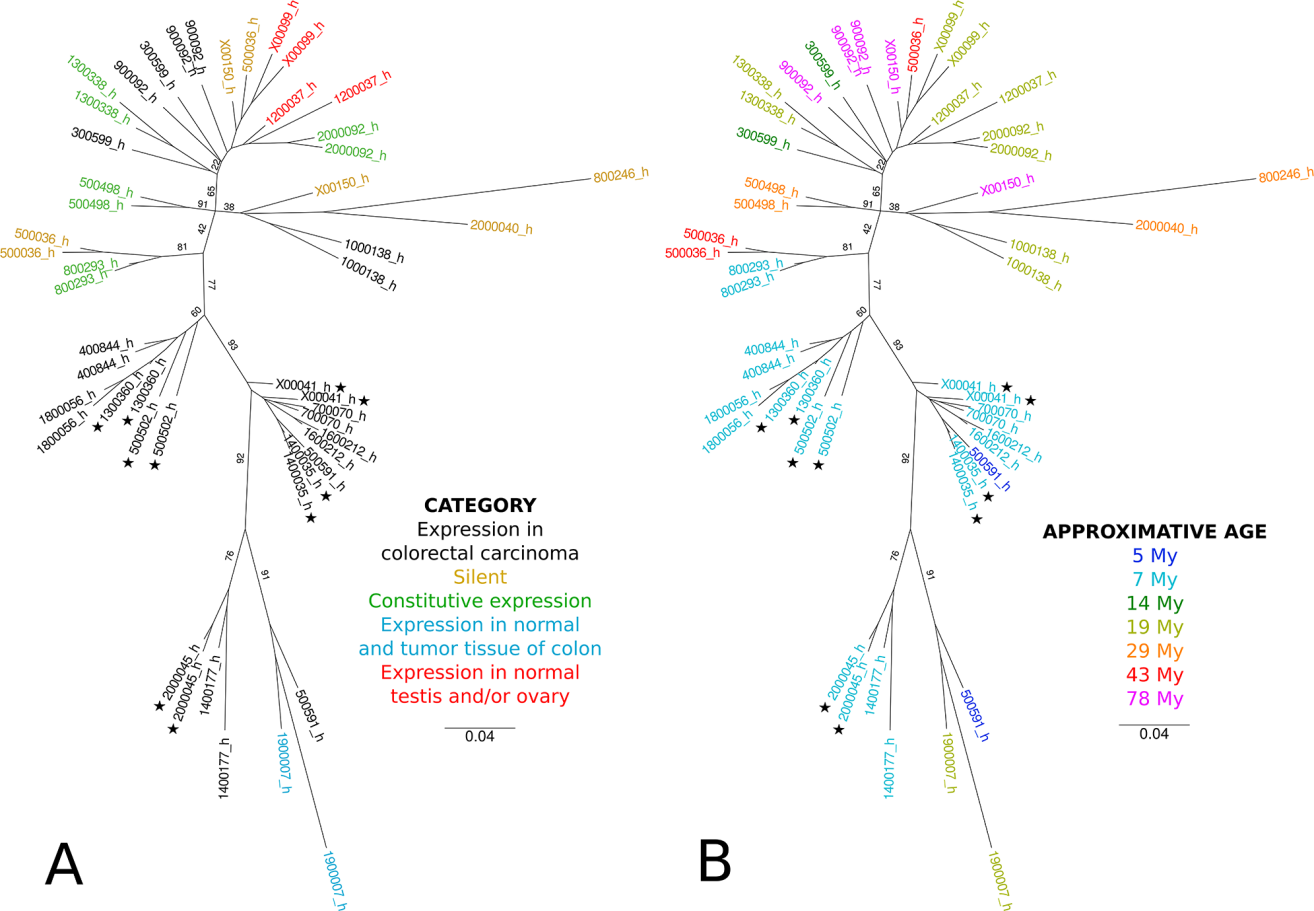
Phylogenetic reconstruction of LTR from CRC-specific, silent, constitutively expressed and gonads-specific loci LTR were aligned using Muscle [46]. Phylogeny was reconstructed by maximum likelihood using PhyML and optimized parameters (see Materials and methods) [47]. Values on branches indicate bootstrap supports (computed on 100 replicates). Stars indicate loci that were validated in the clinical cohort by qRT-PCR. The scale bar represents the average number of substitutions per site. LTR were colored according to **A.** the category of the locus or **B.** according to its approximate age.

The CRC specific HERV-H loci (*n* = 14) were compared to HERV-H sequences found to either be expressed in many tissues (constitutive; *n* = 5), never (silent; *n* = 4) or only in normal testis and/or ovary (gonads; *n* = 2) to potentially identify specific characteristics. General information including category, name, genomic location, domain composition of the sequence and nearest gene can be found in Supplementary table 2. The conservation of the HERV-H elements as well as the time of insertion in primates was deduced from the Ensemble genome multiple alignment (Supplementary Table S3). A phylogeny of the LTR regions was also reconstructed by Maximum Likelihood (Figure 6). Strikingly, most of CRC specific loci (11/14) grouped in distinct clusters in the phylogeny of LTR, compared to loci from other categories (Figure 6A). Using the approximate ages of divergence nodes of the primate phylogenetic tree, the CRC specific HERV-H sequences are significantly younger than the silent or constitutively active ones (CRC vs. silent (*p* = 0.0025), CRC vs. constitutively active (*p* = 0.0148), CRC vs. silent plus constitutively active (*p* = 0.0011); Wilcoxon test; Figure 6B). A less pronounced difference is observed between the age of the silent and constitutively active sequences (silent vs. constitutive (*p* = 0.0312); Wilcoxon test).

The potential functional activity of LTR was investigated by analyzing their overlap with different histone modifications, DNase hypersensitivity sites, as well as with TFBS from the ENCODE database [33]. These overlaps are presented in Supplementary Table S3. While no particular trend could be deduced in terms of TFBS or DNase accessibility, an enrichment of activating histone modifications (e.g. H3k9ac, H3k27ac) for the 5′ LTR of the CRC specific sequences was observed compared to the silent and constitutive loci (*p* = 0.0024). Conversely, almost no repressive histone mark (eg H3k9me3) was observed at the 5′ LTR of the CRC specific sequences. A gene ontology analysis of the genes surrounding CRC specific loci could not detect any significant pathway enrichment (data not shown).

1,000 Genomes Project data [37] were consulted to check for possible structural variants regarding the different HERV-H loci. Notably, four out of the five loci validated on the clinical cohort were found to be conserved in the whole population. One deletion present in only 8 individuals concerned the 500502_h CRC specific locus. However, this deletion could not be associated with any predicted functional effect according to the 1000 Genomes data.

## DISCUSSION

### Clinical validation and signification of HERV-H expression

A HERV-H sequence located at Xp22.3 has repeatedly been described to be expressed in CRC. In small cohorts of 18 to 34 tumor tissue samples, expression is reported in 47% to 95% of analyzed CRC samples [21, 23, 42]. In the current large analysis of clinical samples (*n* = 139 CRC and adjacent normal tissue pairs), we could confirm such expression in half of the tumor samples (70/139). The discrepancy of expression rate with respect to the Asian samples analyzed in Liang et al. [23] may, however, be due to their very small sampling size. Nevertheless, these studies identified overlapping mechanisms regulating the expression of the Xp22.3 locus. Wentzensen et al. showed that the expression correlates with demethylation of the 5′ LTR [21] and the Liang group demonstrated promoter activity of this 5′ LTR [43].

The identified and thoroughly selected HERV-H sequences of the current study included – beside the conversant locus on the X chromosome – novel loci on chromosomes 5, 13, 14, and 20. Expression was restricted to cancerous tissue of the colorectum and present in up to a third of the CRC samples, for the most expressed sequence of these additional loci. Special emphasis was put on common sites of metastasis [41] to complete the view of CRC specificity. HERV-H expression, again, was shown to be strongly CRC specific. Interestingly, the HERV-H colon-tissue positive control appears to be a powerful indicator for the tissue of origin in CRC liver metastases. However, HERV-H Xp22.3 expression was previously described in 40% of gastric, in one of six liver and two of twelve pancreatic carcinomas [21, 23]. This could be due to population size as well as composition or design of primers. Notably, we observed a low expression in pancreatic carcinomas of the loci on chromosome 20 and in one case also the one on chromosome 5. Generally, molecular similarities between pancreatic and colorectal carcinomas, especially with regard to gene expression, have been reported [44].

Additionally, a larger panel of adenomas (*n* = 21) and metastases (*n* = 16) was analyzed for expression of the five HERV-H loci. In this, we could confirm and extend the findings by Alves and co-workers, who found HERV-H Xp22.3 expressed in one adenoma and eight metastases [42]. We found HERV-H Xp22.3 expressed in 29% of adenomas and additionally the locus on chromosome 20 in a third of adenomas. In metastases, especially the locus on chromosome 13 was found present (1300360_h_gag expressed in 11/16). However, the env gene of this particular locus was expressed only marginally in our metastatic samples (1/16). Such a divergence in expression between two genes of the same locus was neither observed in adenomas nor in tumors. Thus, the loss of 1300360_h_env expression may be a CRC liver metastasis specific event and either due to alternative splicing in the metastases or a genomic loss during the metastization process. Overall, we detected transcripts of the five CRC restricted HERV-H loci already present in a third of pre-cancerous adenomas and in up to half of the tumor and metastatic samples. Thus HERV-H expression is generally maintained in all stages of colorectal carcinogenesis.

Bioinformatics analysis of the CRC specific HERV-H sequences in comparison to constitutively active, silent or gonads specific HERV-H loci revealed that the CRC specific HERV-H loci grouped in distinct clusters compared to loci from other categories. Moreover, they are younger in age and possess more activating histone marks on their 5′ LTR. On one hand, the fact that these sequences are rather recent (about 7 million years) and possess transcription enabling histone marks suggests that, from an evolutionary point of view, they are not (yet) as tightly repressed as more ancient (H)ERV. On the other hand, the detected histone marks are similar to the ones observed for the tightly controlled active LTR of the domesticated Syncytin-1 [45] as well as active enhancers [46]. This suggests an independent acquisition or preservation of CRC related TFBS. So far no explanation for heterogeneity in expression in patients could be provided based on comparison of the CRC specific loci with silent and constitutive ones, including SNP in the 1000 Genomes Project (data not shown). In order to decipher CRC specific HERV-H features systematic sequencing of the tumor together with transcriptional activity is required.

### Clinical interpretation

The observed HERV-H expression patterns were assessed with regard to clinical parameters and molecular features of the CRC. A strong correlation of HERV-H expression and MSI as well as lymph node infiltration was observed (*p* < 0.0001 and *p* = 0.0006 respectively).

Clinically, tumors with high instability in microsatellite regions are associated with a better prognosis [47]; caused by the high degree of lymphocyte infiltration due to strong immunogenicity of these tumors. Such immunogenicity results from the appearance of numerous neoantigens due to the inactivation of the mismatch repair system (MMR) – by functional loss of hMLH1 protein due to methylation (in sporadic MSI) and germ line mutations in hMLH1 and other MMR genes (in HNPCC/Lynch syndrome) [48]. To date the known mechanisms MSI positive tumors acquire to evade this cataclysmic immune pressure include downregulation of MHC expression [49] and elevated expression of counter-inhibitory checkpoints [50]. Very recently, A HERV-H derived peptide has been found to add to the arsenal of immune escape mechanisms [51]. Further HERV-triggered immunosuppressive mechanisms would be especially useful for tumors with a high level of immune cell infiltration – as is the case for MSI positive CRC. Mechanistically, we hypothesize that HERV-H ORF interrupted by inactivating mutations might effectively be restored by MSI-induced frameshift mutations [48]. Thus, MSI positive tumor cells expressing such small ORF/peptides would gain a selective advantage. This hypothesis is compatible with the observed increased rather than exclusive HERV-H expression in MSI positive CRC compared to microsatellite stable tumors.

In contrast to the better prognosis connected with MSI, infiltration of tumor cells into lymph nodes represents a feature of more aggressive tumors. However, no correlation with the M status (presence or absence of metastasis at time of resection) was observed. This could hint towards potential involvement of HERV transcripts in early metastasizing processes, facilitating epithelial to mesenchymal transition and preparation of the metastatic niche by exosomes incorporating HERV-H transcripts. Yet, in the subsequent steps of metastasis, such as the reversion of epithelial to mesenchymal transition, HERV-H transcripts may be less important.

### HERV-H as biomarkers for CRC

Stool DNA analyzes detect mutations or methylation patterns within classical cancer genes such as APC, KRAS or TP53. Only recently, a panel combining 7 reference mutations and 4 methylation markers with a sensitivity of 92% for CRC (42% for adenoma) and a specificity of 86% became the first stool-based DNA test receiving FDA approval for CRC screening [52]. Testing for RNA in stool is less-established, but previous studies have documented the potential of stool-based RNA markers, too [53, 54]. A combination of HERV-H transcripts could improve CRC detection. Although preliminary, this approach shows that, by combining the four most expressed HERV-H sequences, the detection of adenocarcinoma could reach 67% sensitivity and 97% specificity on the clinical cohorts. Combining HERV-H Xp22.3 with OPN increases performances up to 82% sensitivity and 99% specificity. Even for pre-cancerous adenomas, the combination of HERV-H Xp22.3 and MMP7 reaches values of 71% and 81%, respectively. Thus, HERV-H might help to further improve existing tests for the detection of pre-cancerous colorectal lesions. Finally, tissue/entity specificity of HERV-H expression may – as opposed to OPN and MMP7 – also provide a handy diagnostic tool for tumors and metastases of unknown origin.

## CONCLUSION

To sum up, this work confirmed the CRC specific expression of HERV-H loci in a large multicentric clinical study. Previously uncharacterized HERV-H sequences were successfully identified by taking advantage of the Affymetrix HERV custom microarray developed in our lab [24]. Herein, for the first time, expression of HERV-H sequences was shown to be associated with the microsatellite status of the tumor and with the nodal status. This allowed discussing a potential involvement of HERV-H in general and especially in MSI-positive colon tumorigenesis and immune evasion. The strong CRC specificity underlines the promising use of HERV-H sequences as a novel class of RNA biomarkers. Together, these results consolidate the rational behind the use of the HERV repertoire for diagnosis and/or therapeutic purposes.

## MATERIALS AND METHODS

### Samples from commercial suppliers

Eighteen matched-pairs of tumor/normal RNA and eleven tumor RNA samples of human colon were purchased from Clinisciences (Nanterre, France). Two additional matched RNA-pairs were obtained from Life Technologies (Carlsbad, USA) and one matched RNA-pair from Agilent Technologies (Santa Clara, USA).

### Samples from the Tumorothèque Champagne-Ardenne (TCA)

Forty matched-pairs of tumor/normal RNA samples of human colon were obtained from the Tumorothèque Champagne-Ardenne (CHU Reims / Institute Jean Godinot, France) as part of a material transfer agreement (signed agreement between BioMérieux SA and TCA). Tumor and normal tissues were characterized by a trained pathologist. RNA extractions were performed using an automated procedure and the quality of RNA was assessed on Experion chips.

### Samples from the department of general surgery of the University of Rostock

Ninety-nine matched-pairs of tumor/normal RNA samples of human colon tissues, plus RNA from the following tissues: sixteen liver metastases of CRC/normal liver, nine adenoma/normal colon, five lung tumor/normal lung, three gastric tumor/normal stomach, four liver tumor/normal liver as well as three pancreatic tumor/normal pancreas were obtained from the Department of General Surgery of the University of Rostock, Germany. Collection of Clinical samples was standardized in the context of the Northern German Tumor Bank for Colorectal Carcinoma and is now performed according to routine SOP [25]. RNA was isolated using the peqGOLD total RNA kit according to the manufacturer's instructions (Peqlab, Erlangen, Germany).

### Samples from the centre hospitalier lyon-sud

Twelve samples from adenoma and peritumoral tissue were obtained from the Tumor Tissue Bank of Hospices Civils de Lyon, supported by National Institute of Cancer (INCa) and French Ministery of Health.

### RNA quality control

RNA quality after transport was assessed using the Bioanalyser 2100 capillary electrophoresis device and RNA Nano Chips kit (both Agilent) prior to cDNA synthesis.

A detailed list of all samples is provided as Supplementary Material (see Supplementary Table S1).

### Ethical considerations for human biological samples

All human tissue specimens used in the present study were obtained in compliance with the ICH-GCP guidelines, European and/or French/German regulations for the use of human biological samples.

For samples provided by the French Centre de Ressources Biologiques of Reims and the tissue Bank of Hospices Civils de Lyon patients were informed and gave their consent prior to any tissue sample conservation and for research use, according to the French Bioethics Law (2004).

For the human tissue specimens obtained from the Surgery Department of the University of Rostock, all procedures were approved by the Ethics Committee of the Medical Faculty at the University of Rostock (Ethikkommission an der Medizinischen Fakultät der Universität Rostock, St.-Georg-Str. 108, 18055 Rostock, Germany; reference number II HV 43/2004) in accordance with guidelines for the use of human material. An informed consent form was obtained in written for all patients.

Procedures for collection, storage and release of tissue samples are in accordance with current recommendations and a quality management program has been developed. All projects submitted to the tissue bank are reviewed and approved by its Scientific Committee, which also verifies their conformity to ethical regulations.

Clinisciences, Life Technologies and Agilent Technologies have signed an agreement to ensure that the tissue samples were obtained in compliance with ICH-GCP standards.

### Primer design and validation

Locus-specific HERV-H PCR primer pairs were designed using Primer3 and the NCBI Primer-BLAST software (http://www.ncbi.nlm.nih.gov/tools/primer-blast) and checked *in silico* at UCSC (http://genome.ucsc.edu). HPLC-purified primers were from Eurogentec (Angers, France). Experimental validations were carried out on human genomic DNA by varying the annealing temperature (Tm) from 55°C to 65°C, and amplification cycles were followed by High Resolution Melting (HRM, Rotor Gene Q; Qiagen, Limburg, Netherlands), gel electrophoresis analysis (Bioanalyzer 2100, Agilent) and product sequencing (GATC Biotech, Konstanz, Germany). Systems meeting the following three criteria (I) one HRM peak, (II) fragment size corresponding to the expected product, and (III) match for the targeted HERV-H sequence after Sanger sequencing, were validated. Primer pairs along with an illustration of the experimental validation scheme (700070_h pol) are summarized in Supplementary Table 2.

### Real-time PCR

Total RNA (100 ng) was DNAse-treated and reverse transcribed (QuantiTec Reverse Transcription Kit, Qiagen). Reverse-transcriptase-free reactions were carried out to verify the absence of contaminating genomic DNA using the TaqMan Gene Expression Assay Human 18S system Hs03003631-g1 and TaqMan Universal PCR kit (both Life Technologies). SYBR green experiments were set up using the Type-it HRM PCR kit in 15 μl final reaction volume with 0.3 μM primers and a 20-fold cDNA dilution. PCR amplifications were carried out in Rotor-disc 100 wrapped discs using a Qiagility robot (all Qiagen). The cDNA amplifications were performed as follows: a 5 min denaturation step at 95°C followed by 45 cycles (95°C for 10s, Tm for 30s, 72°C for 10s) and HRM analysis (from 65°C to 95°C, 0.1°C increments every 2s). All reactions were performed in duplicates. Expression of housekeeping genes G6PD, GAPDH and HPRT was monitored for normalization purposes in the same batch of experiments as the HERV-H targets.

### qRT-PCR data analysis

For each system, the second derivative calculation was used to assess the amplification efficiency (Eff) and relied on the analysis of 45 amplification cycles to ensure optimal curve fitting. Relative Expression (RE) values were defined as Eff^ΔCt^, in which ΔCt = Ct_min-series_ – Ct_sample_. Ct values greater than 33 (for samples from Rostock) or 32 (all other samples) were set at 33 and 32, respectively to avoid over-interpretation of extremely low expression levels. HERV-H RE values were normalized by using the geometric mean of RE values corresponding to G6PD, GAPDH and HPRT. Within a sample series, the lowest normalized RE value was finally arbitrary set at 1 and other values scaled up in order to provide a final relative differential expression view. *T*-test and graphical representations were achieved using GraphPad Prism 5.0 (GraphPad, La Jolla, USA).

### Conservation of the HERV-H loci in primates

Multiple alignment of human sequences of interest were extracted from the Ensembl 100-way multiZ genome alignment (Ensembl75 [26]) using the « Stitch MAF Blocks » utility from the Galaxy server [27–29], for the following primate species: human (*Homo sapiens*, hg19), chimp (*Pan troglodytes*, panTro4), gorilla (*Gorilla gorilla*, gorGor3), orangutan (*Pongo abelii*, ponAbe2), gibbon (*Nomascus leucogenys*, nomLeu3), rhesus macaque (*Macaca mulatta*, rheMac3), crab-eating macaque (*Macaca fascicularis*, macFas5), hamadryas baboon (*Papio hamadryas*, papHam1), vervet monkey (*Chlorocebus sabaeus*, chlSab1), marmoset (*Callithrix jacchus*, calJac3), squirrel monkey (*Saimiri boliviensis*, saiBol1), and bushbaby (*Otolemur garnetti*, otoGar3). The orthology of aligned sequences was further verified: (I) by looking for orthologous genes in the environment of the sequences in the different species (using orthology links defined by Ensembl in the file ancGenes.Euteleostomi.list.bz2), and (II) by checking the similarity of directly flanking sequences between the different species. After this cleaning step, the age of each HERV-H locus studied was approximated to the age of the split between human and most distant species where the locus was found. These node ages were extracted from the works of Steiper & Young 2006 [30] and Locke 2011 [31]. The distribution of ages between the different categories of loci was tested using a Wilcoxon test and implemented in R [32].

### Overlap with histone modifications, DNase hypersensitivity data and transcription factor binding sites (TFBS)

Histone modification data were downloaded from the ENCODE project [33] (histone peaks available here: http://ftp.ebi.ac.uk/pub/databases/ensembl/encode/integration_data_jan2011/byDataType/peaks/jan2011/histone_macs/optimal/hub), for all cell lines available and for following marks: H2az, H3k4me1, H3k4me2, H3k4me3, H3k9ac, H3k27ac, H3k79me2, H3k9me3, H3k27me3, H3k9me1, H3k36me3 and H4k20me1. For each mark, overlaps of the LTR with data from the different cell lines were computed using the Bedtools *intersect* utility [34]; a mean score was then obtained by averaging the overlaps measured in the different cell lines.

### DNase hypersensitive sites were also retrieved from ENCODE data [33] (DNAse clusters available here

http://hgdownload.cse.ucsc.edu/goldenPath/hg19/encodeDCC/wgEncodeRegDnaseClustered/). As for histone marks, overlaps of these DNase clusters with LTR were obtained using the Bedtools *intersect* utility [34].

Overlap of HERV-H loci with TFBS data from ENCODE was directly retrieved from the UCSC genome browser [35] (integrated regulation track “Transcription Factor ChIP-seq (161 factors)) [36].

### Overlap with structural variants from the 1,000 genome project data

Possible indels involving, at least partially, the different HERV-H elements were sought after in the 1,000 Genome Project data [37] accessed via the Ensembl genome browser [26].

### Gene ontology analysis of surrounding genes

A possible functional enrichment of genes found in the vicinity of the CRC specific HERV-H loci (in 100, 500 and 1,000 kb windows centered on these loci respectively) compared to all human genes was tested using GOrilla [38].

### LTR phylogenetic reconstruction

LTR sequences were aligned using Muscle [39] with default parameters. Molecular phylogeny was reconstructed by maximum likelihood using PhyML [40] with 100 bootstrap replicates, optimized nucleotide equilibrium frequencies, optimized invariable sites and SPR tree searching operations.

## SUPPLEMENTARY FIGURES AND TABLES








