# Supplementary material for: Expression of young HERV-H loci in the course of colorectal carcinoma and correlation with molecular subtypes
Source: Oncotarget. 2015 Oct 23;6(37):40095–111. doi: 10.18632/oncotarget.5539 (PMC4741882; doi:10.18632/oncotarget.5539)
Supplement: Supplementary file 1 [file oncotarget-06-40095-s001.pdf]

SUPPLEMENTARY FIGURES AND TABLES

Selection and conception of PCR systems

HERV targets

locus id	locus localization (NCBI36/hg18)	PCR systems					Primer sequences
		5'LTR	gag	pol	env	3'LTR	
300599_h	chr 3: 167701085-167706900		x				TCTCTGTTCCCAATGCAACTCGT<br>GAGGGCCAGTCAGGGAATGAAACTG
400844_h	chr 4: 183972457-183977066				x		TGTTTTTCGCCTTCTCATATTCCAT<br>TGTGGTAAGGGGTGATATTGTGG
500502_h	chr 5: 135904531-135912105					x	CCCAGATGGCCTGAAGTAACTGA<br>AGCCAGGAGAACAATTCACAGGGTT
500591_h	chr 5: 179775292-179781337			x			CCTAGTCTCTGTGCCCAATGCAA<br>AACTGTAAGCCAGAGCAGGTGTG
700070_h	chr 7: 26030345-26035880			x			ATACAGTCTGATAACGGACCAGC<br>ATTGTCAGTCCTTTTAAGTTGGTGG
900092_h	chr 9: 25658927-25666384		x				ATAGGCAAACGGTCTGAGATGCC<br>GACCAGGTTTCAGGAGGGGAGGT
1000138_h	chr 10: 45388242-45391541		x				GCCCCGCCACCCTACAATCC<br>TTGCTGGGCAGGTGGGGGA
1300360_h	chr 13: 108715439-108721465		x	x	x		AGTGCAACTCATTCTGAATCTTCCT<br>CACAGAACGAAACTGTAAGCCAG<br>AATTAGCTTTACTCCACATGCCC<br>AAGGGATATAAACTGAAAAACTGCT<br>CTGAACCTCCTTAGGCATTCTCT<br>GTGACATTGAGGGGGTTGTTAGAAG
1400035_h	chr 14: 30785319-30790095		x				CCCAAGTGTCGCTGAGTCTTTCT<br>GATTACAGGGTGCAGGAGCAGAG
1400177_h	chr 14: 73239795-73245370		x	x	x		TTCCTAGTCTTTGTCCCCAATGCAA<br>AGGTGTGAGGAGGCGAGGTGATAAA<br>CACGGTAGAAGGTAGTAAGCGCGTC<br>GCGGCAATGAGATGTGGCTGTAGTC<br>TCCAAAACCATATGCAGTCCATCAC<br>AGCTGAAGGGAGGTCTTGTGGTAAG
1600212_h	chr 16: 84869202-84872386			x			CCTTCGCGTCTTTCTTTCATATCCC<br>TGAACTAACCTGTAAGCCCTGTC
1800056_h	chr 18: 24526989-24532843		x				CCAGGCATTCTTTCACACATCAG<br>AGGGATACTCATGGAACGAAATTGT
2000045_h	chr 20: 19680691-19685420		x	x			CCCAAGCGGCGCTGAGTCTT<br>TGGGATGAAGGGAGGGGAGGC<br>TACAGCATGGGCACCTATAAACTCT<br>TAAGTGAAGGCAAAGAGAGGCTGGG
X00041_h	chr X: 4468515-4474361		x				CAGGCGTTGCTGAGTGTGTCTAATC<br>TGGAGCCTGAGGAAGAATTGGGACC
(-) 1900006_h	chr 19: 5499587-5504223				x		CCACCGAGGCCTTGACTGACT<br>GGGAGGGCCCAGGACATCCAA
(+) 1900007_h	chr 19: 5797895-5801213	x					ATATCCCCTACGACCGGCTCATATA<br>TGGGGCAGAAACAGATCACAATGGT

MMP7	AGATGTGGAGTGCCAGATGT; TAGACTGCTACCATCCGTCC
OPN	TGGAAGTTCTGAGGAAAAGCAG; GGCTTTCGTTGGACTTACTTG
GAPDH	GAAGGTGAAGGTCGGAGTC; GAAGATGGTGATGGGATTTC
G6PD	TGCAGATGCTGTGTCTGG; CGTACTGGCCCAGGACC
HPRT	GTGATGATGAACCAGGTTATGACCTTG; CTACAGTCATAGGAATGGATCTATCAC

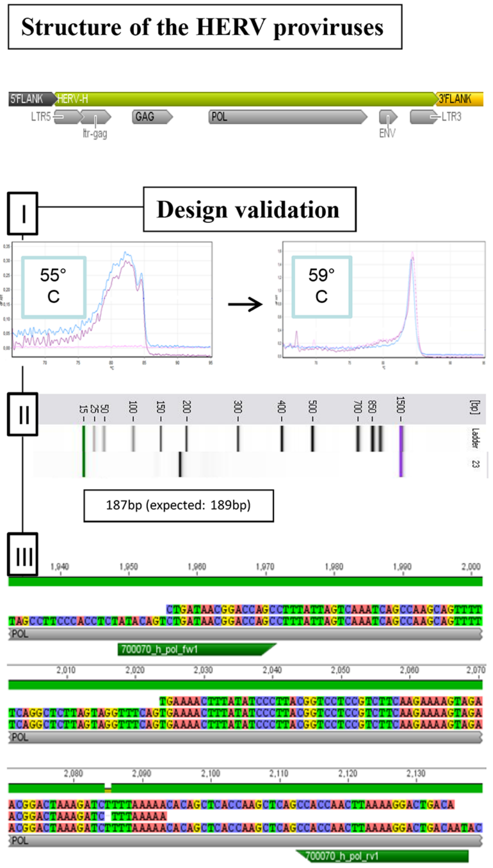

Supplementary Figure S1: Selection and conception of HERV-H locus-specific PCR systems. Starting from annotated HERV-H proviruses (5′LTR, gag, pol, env and 3′LTR), locus-specific PCR primers were designed (left part, 'X' means a PCR system has been designed within the region) and then experimentally validated (right part). The validation, exemplified through the polymerase (pol) region of the HERV-H locus 700070, included **I.** the search for an optimal PCR Tm temperature using HRM, **II.** a gel electrophoresis analysis and **III.** a double Sanger sequencing using the forward (700070_h_pol_fw1) and the reverse (700070_h_pol_rv1) PCR primers. The PCR primers sequences, including controls, are given 5′ to 3′.

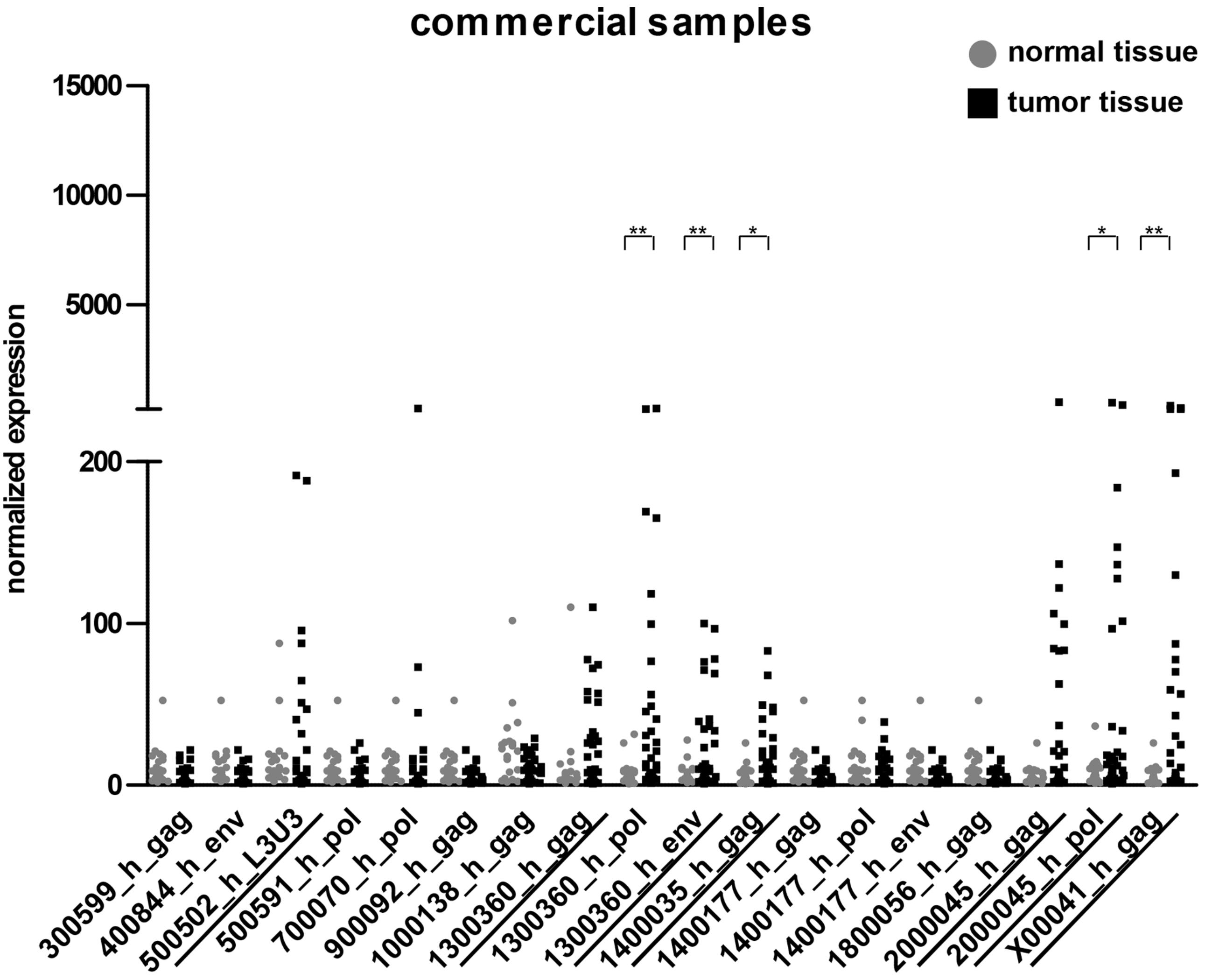

Supplementary Figure S2: HERV-H expression in commercial samples. The expression of 14 HERV-H loci (represented by 19 qRT-PCR systems) for CRC (black squares) and corresponding normal (grey dots) tissue is depicted in the dot plot. Statistically significant differences in expression between normal and tumorous tissue are indicated by stars (**p* < 0.05, ***p* < 0.01, ****p* < 0.001, *t*-test). For better interpretation of the results, controls for tissue specificity (1900006_h_env = negative control, 1900007_h_L3U3 = positive control) and cancerous origin (MMP7 and OPN) were added and are indicated by the horizontal bar. Sequences that were further analyzed in subsequent experiments are encircled by a black line.

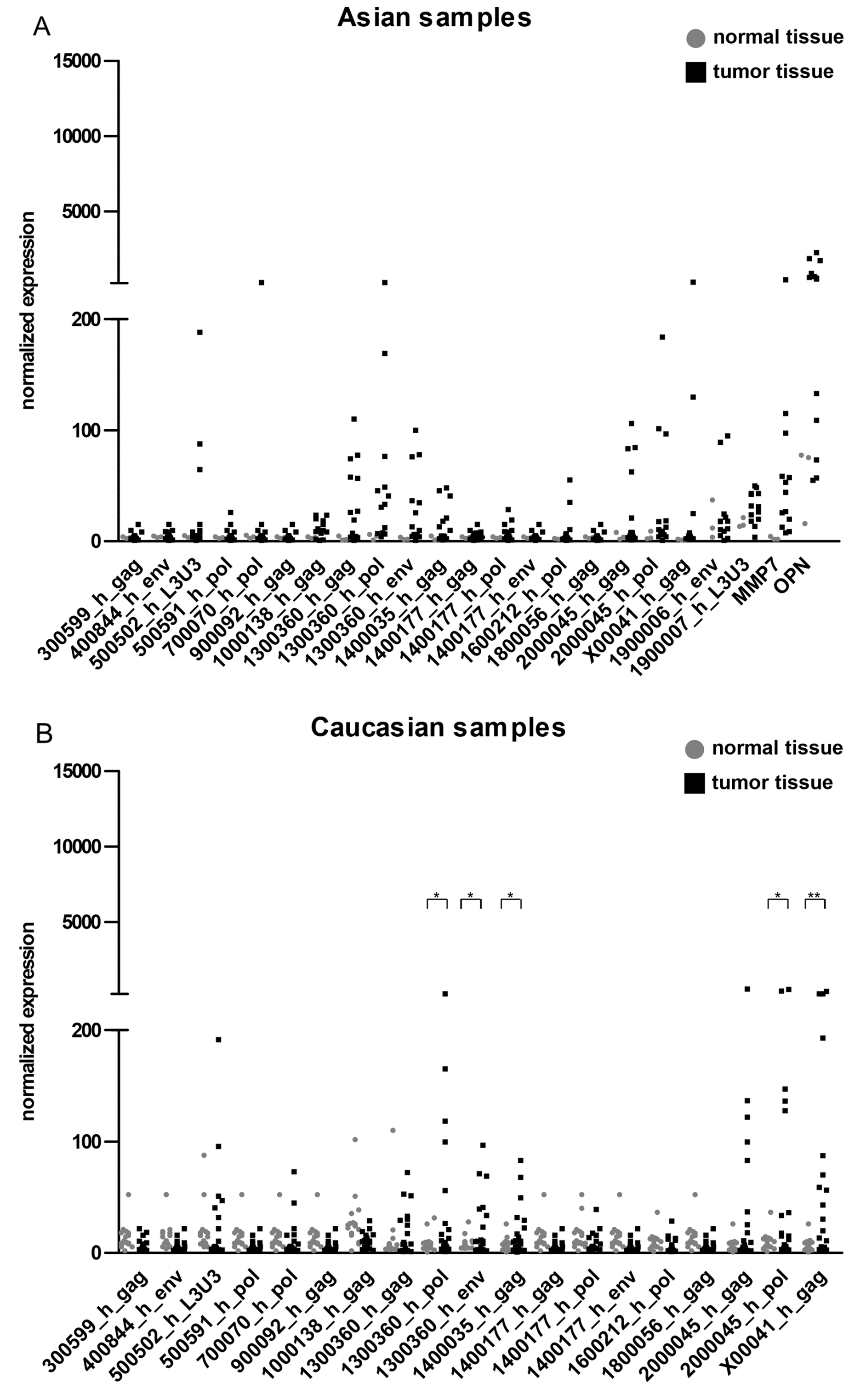

Supplementary Figure S3: Distinction of samples from Asian and Caucasian populations. The expression of 14 HERV-H sequences for **A.** Asian and **B.** Caucasian populations for CRC (black squares) and corresponding normal (grey dots) tissue is depicted in the dot plot. Statistically significant differences in expression between normal and tumorous tissue are indicated by stars (*$p < 0.05$, **$p < 0.01$, ***$p < 0.001$, *t*-test). For better interpretation of the results, controls for tissue specificity (1900006_h_env = negative control, 1900007_h_L3U3 = positive control) and cancerous origin (MMP7 and OPN) were added and are indicated by the horizontal bar.

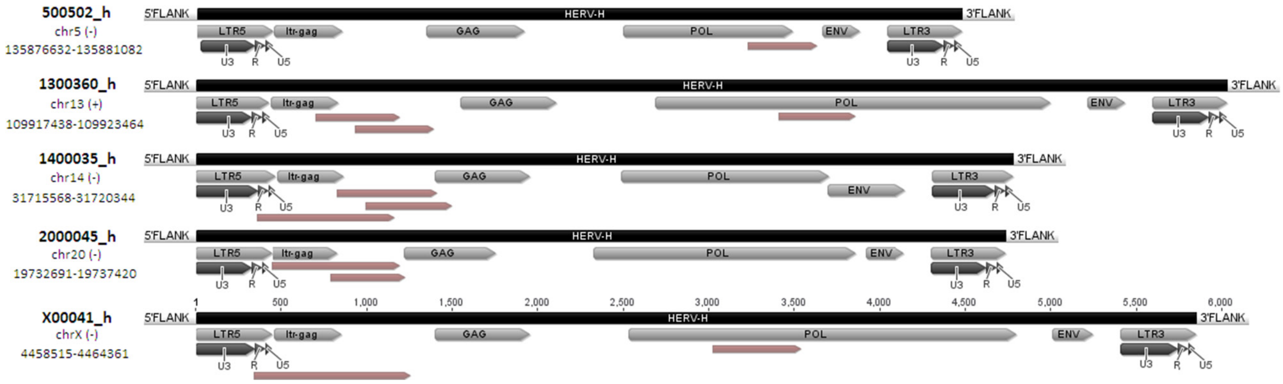

Supplementary Figure S4: HERV-H sequence compositions. Structures of the five HERV-H elements validated on the clinical cohort are given. For each HERV-H sequence, genomic information for chromosome (chr), strand (+/–), start and end positions (hg19) are indicated on the left side. The corresponding HERV-H sequences are depicted along with their functional annotations LTR, ltr-gag, gag, pol and env, which were attributed by homology with a reference HERV-H provirus ((-)chr2: 166564060–166572708). Additional U3, R and U5 sub-regions are given for LTR. Brown arrows indicate ORF predictions based on the standard genetic code and minimum ORF size set to 400nt.

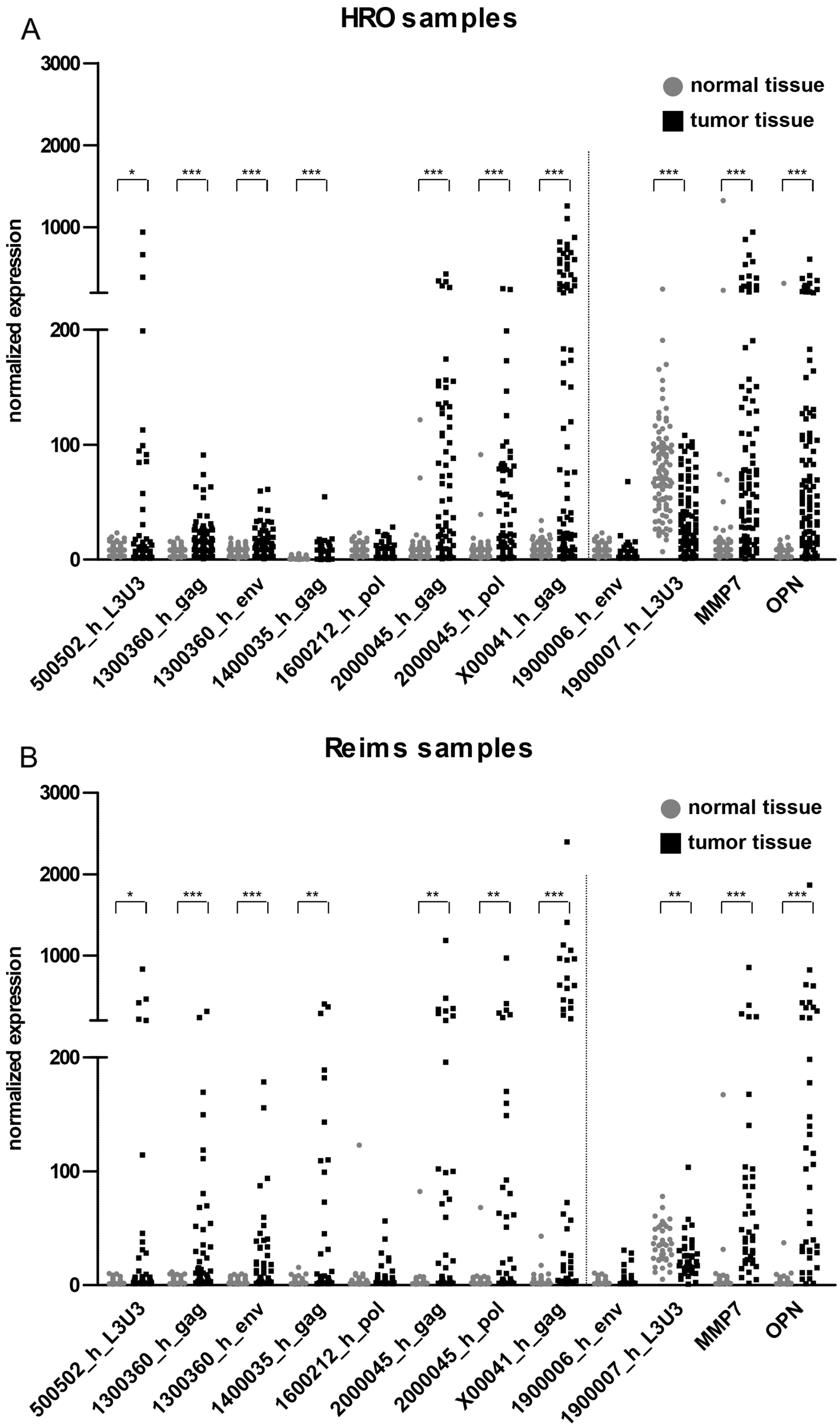

Supplementary Figure S5: Distinction of samples from biobanks of Rostock and Reims. The expression of five HERV-H sequences for samples from the biobanks of **A.** Rostock (HRO) and **B.** Reims containing CRC (black squares) and corresponding normal (grey dots) tissue is depicted in the dot plot. Statistically significant differences in expression between normal and tumorous tissue are indicated by stars (**p* < 0.05, ***p* < 0.01, ****p* < 0.001, *t*-test). For better interpretation of the results, controls for tissue specificity (1900006_h_env = negative control, 1900007_h_L3U3 = positive control) and cancerous origin (MMP7 and OPN) were added and are separated from the HERV-H sequences by the dotted line.

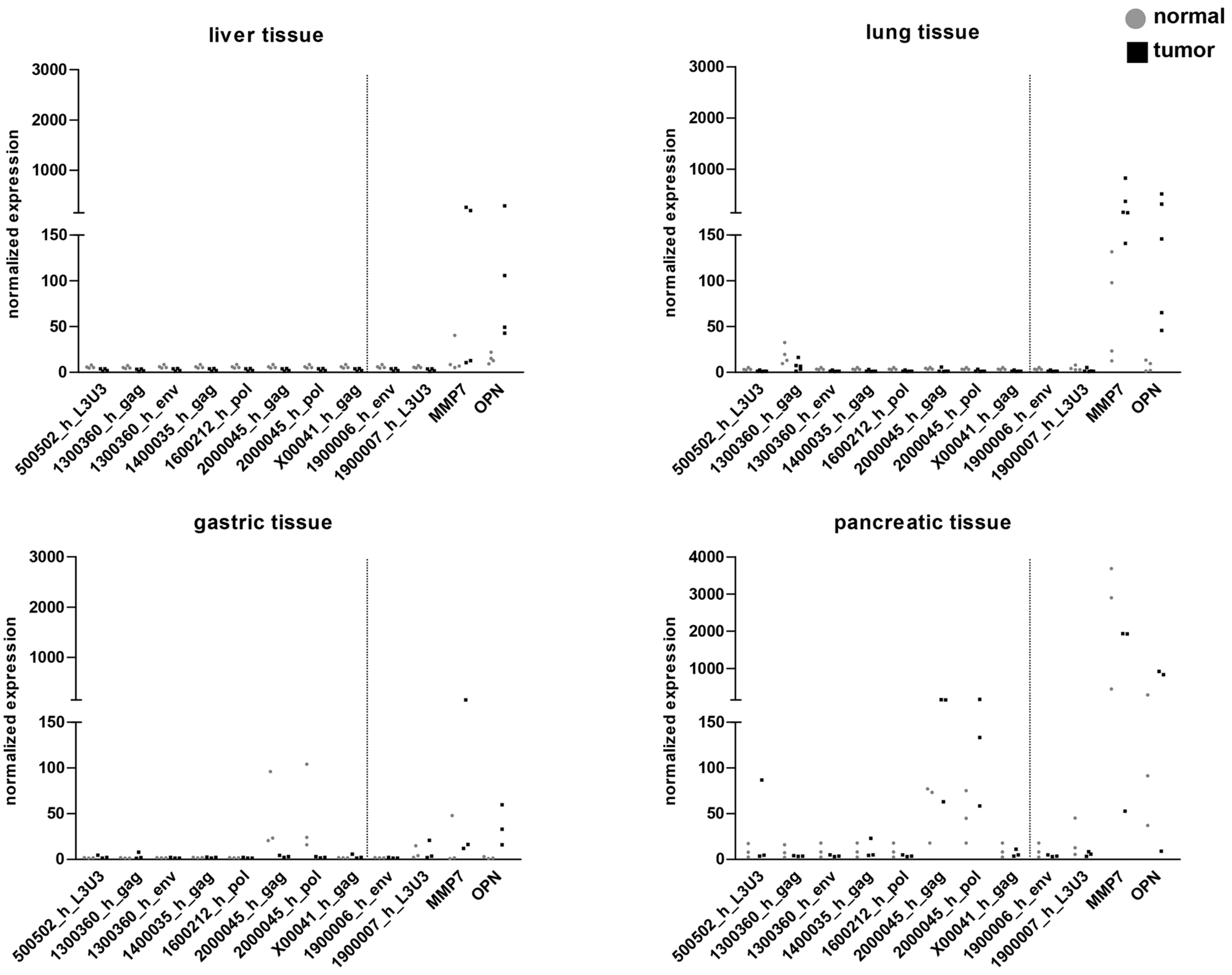

Supplementary Figure S6: HERV-H expression in tissue of common sites for metastasis. The expression of five HERV-H sequences for cancerous (black square) and normal (grey dot) tissue having common sites of metastasis with CRC (liver, lung, stomach and pancreas) is depicted in the dot plot. For better interpretation of the results, controls for tissue specificity (1900006_h_env = negative control, 1900007_h_L3U3 = positive control) and cancerous origin (MMP7 and OPN) were added and are separated from the HERV-H sequences' results by the dotted line.

Supplementary Table S1: Sample overview (See Supplementary File_S1)

The table summarizes the information on sample type (N: normal tissue, A: adenoma, Tu: tumor, Met: metastasis), patients' age (in years at time of resection), gender (M: male, F: female) and ethnicity as well as information on tumor localization, TNM classification, grading, microsatellite (MS) status (MSS: microsatellite stable, MSI: microsatellite instable), common mutations of the tumors (TP53, APC, KRAS, BRAF) and quality of sample (RIN, RNA integrity number) as well as the supplier; n.a. = not assessed

Supplementary Table S2: Structural characteristics of the different loci analyzed (See Supplementary File_S2)

The table lists the information on the assigned category, genomic coordinates, name, size, presence of Target Site Duplications (TSD), length and identity of LTRs, domains composition and closest gene of the HERV-H sequences.

Supplementary Table S3: Evolutionary and functional characteristics of the different loci analyzed (See Supplementary File_S3)

The table lists the information on the assigned category, name, conservation in the primate genome, structural variants from the 1,000 Genome Project, DNase accessibility (ENCODE DNAse clusters) and histone marks (active of repressive) of the HERV-H sequences. The overlap with these different functional annotations was performed as described in Materials and Methods. H3k9me1, H3k36me3 and H4k20me1 modifications, which presented no overlap, were dismissed.
