# Supplementary material for: Expression of young HERV-H loci in the course of colorectal carcinoma and correlation with molecular subtypes
Source: Oncotarget. 2015 Oct 23;6(37):40095–111. doi: 10.18632/oncotarget.5539 (PMC4741882; doi:10.18632/oncotarget.5539)
Supplement: Supplementary file 2 [file oncotarget-06-40095-s002.doc]

| **Sample ID** | **tissue type** | **Age** | **Gender** | **Ethnicity** | **localization** | **TNM** | **Grading** | **MS status** | **mutations** | **RIN** | **Supplier** |
| --- | --- | --- | --- | --- | --- | --- | --- | --- | --- | --- | --- |
| Ct1 / Cn1 | Tu / N | 62 | F | Asian | Colon | n.a. | n.a. | n.a. | n.a. | 8.3 / 7.5 | Clinisciences |
| Ct2 / Cn2 | Tu / N | 68 | F | Asian | Colon | n.a. | n.a. | n.a. | n.a. | 7.3 / 6.6 | Clinisciences |
| Ct3 / Cn3 | Tu / N | 62 | F | Asian | Colon | n.a. | n.a. | n.a. | n.a. | 7.9 / 7.9 | Clinisciences |
| T65 / N64 | Tu / N | 74 | M | Caucasian | Colon | n.a. | n.a. | n.a. | n.a. | n.a. | Ambion |
| T94 / N93 | Tu / N | 70 | F | Caucasian | Colon | n.a. | n.a. | n.a. | n.a. | n.a. | Ambion |
| St1 / Sn2 | Tu / N | n.a. | n.a. | n.a. | Colon | n.a. | n.a. | n.a. | n.a. | n.a. | Stratagene |
| CC1 | Tu | 73 | M | Asian | Colon | n.a. | n.a. | n.a. | n.a. | n.a. | Clinisciences |
| CC2 | Tu | 70 | M | Asian | Colon | n.a. | n.a. | n.a. | n.a. | n.a. | Clinisciences |
| CC3 | Tu | 70 | M | Asian | Colon | n.a. | n.a. | n.a. | n.a. | n.a. | Clinisciences |
| CC4 | Tu | 79 | M | Asian | Colon | n.a. | n.a. | n.a. | n.a. | n.a. | Clinisciences |
| CC5 | Tu | 53 | M | Asian | Colon | n.a. | n.a. | n.a. | n.a. | n.a. | Clinisciences |
| CC6 | Tu | 68 | M | Asian | Colon | n.a. | n.a. | n.a. | n.a. | n.a. | Clinisciences |
| CC7 | Tu | 60 | M | Asian | Colon | n.a. | n.a. | n.a. | n.a. | n.a. | Clinisciences |
| CC8 | Tu | 47 | M | Asian | Colon | n.a. | n.a. | n.a. | n.a. | n.a. | Clinisciences |
| CC9 | Tu | 61 | M | Asian | Colon | n.a. | n.a. | n.a. | n.a. | n.a. | Clinisciences |
| CC10 | Tu | 50 | M | Asian | Colon | n.a. | n.a. | n.a. | n.a. | n.a. | Clinisciences |
| CC11 | Tu | 70 | F | Caucasian | Transverse | T3N0M | G2 | n.a. | n.a. | n.a. | Clinisciences |
| CC12 / CN12 | Tu / N | 60 | F | Caucasian | Coecum | T4N2 | G1 | n.a. | n.a. | n.a. | Clinisciences |
| CC13 / CN13 | Tu / N | 65 | M | Caucasian | Ascending | T3N0 | G1 | n.a. | n.a. | n.a. | Clinisciences |
| CC14 / CN14 | Tu / N | 58 | M | Caucasian | Sigmoid | T3N2 | G1 | n.a. | n.a. | n.a. | Clinisciences |
| CC15 / CN15 | Tu / N | 78 | M | Caucasian | Sigmoid | T3N0 | G2 | n.a. | n.a. | n.a. | Clinisciences |
| CC16 / CN16 | Tu / N | 50 | M | Caucasian | Rectum | T2N0 | G2 | n.a. | n.a. | n.a. | Clinisciences |
| CC17 / CN17 | Tu / N | 80 | M | Caucasian | Recto-sigmoid | T4N0 | G2 | n.a. | n.a. | n.a. | Clinisciences |
| CC18 / CN18 | Tu / N | 38 | M | Caucasian | Recto-sigmoid | T3N2 | G2 | n.a. | n.a. | n.a. | Clinisciences |
| CC19 / CN19 | Tu / N | 78 | M | Caucasian | Sigmoid | T3N0 | G1 | n.a. | n.a. | n.a. | Clinisciences |
| CC20 / CN20 | Tu / N | 80 | M | Caucasian | Transverse | T3N0 | G2 | n.a. | n.a. | n.a. | Clinisciences |
| CC21 / CN21 | Tu / N | 89 | F | Caucasian | Descending | T3N0 | G3 | n.a. | n.a. | n.a. | Clinisciences |
| CC22 / CN22 | Tu / N | 67 | F | Caucasian | Ascending | T4N2 | G1 | n.a. | n.a. | n.a. | Clinisciences |
| CC23 / CN23 | Tu / N | 63 | F | Caucasian | Descending | T3N0 | G2 | n.a. | n.a. | n.a. | Clinisciences |
| CC24 / CN24 | Tu / N | 64 | M | Caucasian | Descending | T4N2 | G1 | n.a. | n.a. | n.a. | Clinisciences |
| CC25 / CN25 | Tu / N | 89 | F | Caucasian | Ascending | T3N1 | G3 | n.a. | n.a. | n.a. | Clinisciences |
| CC26 / CN26 | Tu / N | 37 | F | Caucasian | Transverse | T4N2 | G3 | n.a. | n.a. | n.a. | Clinisciences |
| T10T115 / S10T115 | Tu / N | 68 | M | Caucasian | Colon | T3N0 | n.a. | n.a. | n.a. | 8.0 / 8.2 | Reims |
| T10T123 / S10T123 | Tu / N | 78 | M | Caucasian | Colon | T4N2 | n.a. | MSS | wt | 8.8 / 8.2 | Reims |
| T10T156 / S10T156 | Tu / N | 76 | M | Caucasian | Sigmoid | T3N1 | n.a. | n.a. | KRAS | 6.6 / 8.2 | Reims |
| T11T175 / S10T175 | Tu / N | 79 | F | Caucasian | Colon | T3N1 | n.a. | MSI | n.a. | 8.6 / 7.0 | Reims |
| T10T310 / S10T310 | Tu / N | 51 | M | Caucasian | Colon | T3N1 | n.a. | MSS | Wt | 7.4 / 7.7 | Reims |
| T10T320 / S10T320 | Tu / N | 62 | F | Caucasian | Colon | T4N0 | n.a. | n.a. | Wt | 7.7 / 7.5 | Reims |
| T10T378 / S10T378 | Tu / N | 49 | M | Caucasian | Colon | T4N1 | n.a. | MSS | KRAS | 8.9 / 8.2 | Reims |
| T10T390 / S10T390 | Tu / N | 67 | M | Caucasian | Colon | T3N0M1 | n.a. | n.a. | Wt | 7.6 / 8.7 | Reims |
| T10T392 / S10T392 | Tu / N | 53 | M | Caucasian | Rectum | T3N0 | n.a. | MSS | Wt | 7.6 / 7.7 | Reims |
| T10T395 / S10T395 | Tu / N | 63 | M | Caucasian | Colon | T3N0 | n.a. | n.a. | n.a. | 8.1 / 8.5 | Reims |
| T10T432 / S10T432 | Tu / N | 68 | F | Caucasian | Colon | T3N0 | n.a. | n.a. | n.a. | 8.3 / 8.2 | Reims |
| T10T451 / S10T451 | Tu / N | 86 | M | Caucasian | Colon | T3N0 | n.a. | n.a. | n.a. | 7.9 / 7.8 | Reims |
| T10T490 / S10T490 | Tu / N | 54 | M | Caucasian | Ascending | T4N0 | n.a. | MSI | Wt | 7.0 / 7.9 | Reims |
| T10T526 / S10T526 | Tu / N | 63 | F | Caucasian | Colon | T2N0 | n.a. | n.a. | Wt | 8.2 /8.7 | Reims |
| T11T011 / S11T011 | Tu / N | 50 | M | Caucasian | Colon | T3N2 | n.a. | MSS | n.a. | 7.8 / 9.5 | Reims |
| T11T018 / S11T018 | Tu / N | 79 | F | Caucasian | Colon | T3N1 | n.a. | MSI | BRAF | 8.3 / 8.3 | Reims |
| T11T023 / S11T023 | Tu / N | 64 | F | Caucasian | Sigmoid | T3N0 | n.a. | MSS | n.a. | 8.9 / 7.4 | Reims |
| T11T035 / S11T035 | Tu / N | 72 | M | Caucasian | Colon | T3N0 | n.a. | n.a. | n.a. | 9.1 / 8.6 | Reims |
| T11T053 / S11T053 | Tu / N | 69 | F | Caucasian | Colon | T3N2 | n.a. | n.a. | n.a. | 8.9 / 7.0 | Reims |
| T11T252 / S11T252 | Tu / N | 75 | M | Caucasian | Colon | T3N1 | n.a. | n.a. | n.a. | 8.2 / 6.3 | Reims |
| T11T267 / S11T267 | Tu / N | 81 | M | Caucasian | Sigmoid | T4N1 | n.a. | MSS | n.a. | 6.9 / 7.6 | Reims |
| T11T390 / S11T390 | Tu / N | 87 | F | Caucasian | Colon | T3N1 | n.a. | n.a. | n.a. | 7.7 / 7.6 | Reims |
| T11T391 / S11T391 | Tu / N | 71 | M | Caucasian | Sigmoid | T4 | n.a. | n.a. | n.a. | 7.8 / 7.3 | Reims |
| T11T410 / S11T410 | Tu / N | 79 | F | Caucasian | Colon | T3N0 | n.a. | n.a. | n.a. | 8.7 / 7.6 | Reims |
| T11T411 / S11T411 | Tu / N | 74 | M | Caucasian | Colon | T4N0 | n.a. | MSS | KRAS | 7.6 / 7.5 | Reims |
| T211T414 / S111T414 | Tu / N | 78 | F | Caucasian | Colon | T3N1M1 | n.a. | MSS | n.a. | 7.7 / 7.7 | Reims |
| T11T417 / S11T417 | Tu / N | 80 | F | Caucasian | Sigmoid | T4N1 | n.a. | n.a. | n.a. | 7.8 / 7.9 | Reims |
| T11T431 / S11T431 | Tu / N | 51 | M | Caucasian | Colon | T3N1 | n.a. | MSI | Wt | 7.6 / 7.6 | Reims |
| T11T432 / S11T432 | Tu / N | 63 | F | Caucasian | Sigmoid | T3N0 | n.a. | MSS | n.a. | 8.2 / 8.2 | Reims |
| T11T445 / S11T445 | Tu / N | 74 | F | Caucasian | Colon | T4N0 | n.a. | MSI | BRAF | 7.3 / 7.3 | Reims |
| T11T506 / S11T506 | Tu / N | 74 | M | Caucasian | Colon | T4N0 | n.a. | MSS | n.a. | 8.6 / 7.8 | Reims |
| T11T534 / S11T534 | Tu / N | 76 | M | Caucasian | Colon | T4N2M0 | n.a. | n.a. | Wt | 7.4 / 7.6 | Reims |
| T11T545 / S11T545 | Tu / N | 67 | F | Caucasian | Sigmoid | T4N1 | n.a. | n.a. | KRAS | 6.9 / 7.3 | Reims |
| T11T654 / S11T654 | Tu / N | 75 | F | Caucasian | Colon | T3N2 | n.a. | n.a. | Wt | 7.9 / 8.1 | Reims |
| T11T659 / S11T659 | Tu / N | 77 | F | Caucasian | Colon | T3N0 | n.a. | MSS | n.a. | 7.6 / 9.5 | Reims |
| T12T111 / S12T111 | Tu / N | 71 | M | Caucasian | Colon | T3N0 | n.a. | MSS | n.a. | 8.4 / 8.1 | Reims |
| T12T200 / S12T200 | Tu / N | 81 | F | Caucasian | Colon | T3N1 | n.a. | n.a. | n.a. | 7.3 / 7.5 | Reims |
| T12T220 / S12T220 | Tu / N | 80 | M | Caucasian | Colon | T3N0 | n.a. | MSS | n.a. | 7.6 / 7.2 | Reims |
| T12T264 / S12T264 | Tu / N | 53 | F | Caucasian | Colon | T3N0 | n.a. | MSS | n.a. | 7.8 / 8.0 | Reims |
| T12T303 / S12T303 | Tu / N | 50 | F | Caucasian | Colon | T3N0 | n.a. | MSS | n.a. | 8.9 / 8.1 | Reims |
| HROC80 | Tu / N | 72 | M | Caucasian | Coecum | T3N2 | G2 | MSS | KRAS, TP53 | 7.6 / 3.9 | Rostock |
| HROC81 | Tu / N | 21 | F | Caucasian | Ascending | T4N0M0 | G3 | MSS | wt | 8.5 / 7.9 | Rostock |
| HROC82 | Tu / N | 62 | M | Caucasian | Transverse | T3N0 | G2 | MSS | TP53 | 7.9 / 7.3 | Rostock |
| HROC84 | Tu / N | 88 | F | Caucasian | Sigmoid | T3N0M0 | G2 | MSS | wt | 7.9 / 9.1 | Rostock |
| HROC86 | Tu / N | 79 | F | Caucasian | Sigmoid | T3N1M0 | G2 | MSS | KRAS, TP53, APC | 9.6 / 9.5 | Rostock |
| HROC87 | Tu / N | 76 | F | Caucasian | Ascending | T3N0M0 | G3 | MSI | BRAF, TP53 | 8.9 / 6.4 | Rostock |
| HROC88 | Tu / N | 69 | F | Caucasian | Ascending | T3N1M0 | G2 | MSS | wt | 8.3 / 7.9 | Rostock |
| HROC89 | Tu / N | 80 | M | Caucasian | Rectum | T3N0M1 | G2 | MSS | KRAS, TP53, APC | n.a. / n.a. | Rostock |
| HROC90 | Tu / N | 84 | F | Caucasian | Rectum | T2N0M0 | G2 | MSS | wt | 7.9 / 7.9 | Rostock |
| HROC92 | Tu / N | 60 | M | Caucasian | Ascending | T3N1M1 | G2 | MSS | TP53, APC | 5.8 / 5.1 | Rostock |
| HROC93 | Tu / N | 72 | M | Caucasian | Hepatic flexure | T3N0M0 | G3 | MSS | wt | 8.4 / n.a. | Rostock |
| HROC94 | Tu / N | 47 | F | Caucasian | Coecum | T4N2M1 | G1 | MSS | wt | 8.5 / 5.4 | Rostock |
| HROC95 | Tu / N | 74 | M | Caucasian | Sigmoid | T2N0M0 | G2 | MSS | TP53, APC | n.a. / 6.8 | Rostock |
| HROC96 | Tu / N | 63 | F | Caucasian | Sigmoid | T4N0M0 | G2 | MSS | KRAS | 8.5 / 6.0 | Rostock |
| HROC97 | Tu / N | 88 | M | Caucasian | Rectum | T2N1M0 | G2 | MSS | KRAS | 6.2 / 5.1 | Rostock |
| HROC98 | Tu / N | 66 | F | Caucasian | Ascending | T4N2M1 | G3 | MSS | KRAS | n.a. / 8.0 | Rostock |
| HROC99 | Tu / N | 60 | M | Caucasian | Rectum | T3N2M0 | G2 | MSS | KRAS | n.a. / n.a. | Rostock |
| HROC100 | Tu / N | 82 | M | Caucasian | Sigmoid | T2N0M0 | G2 | MSS | wt | 8.7 / 9.0 | Rostock |
| HROC101 | Tu / N | 78 | F | Caucasian | Sigmoid | T3N2M1 | G2 | MSS | KRAS | 8.4 / 7.7 | Rostock |
| HROC102 | Tu / N | 81 | F | Caucasian | Sigmoid | T2N0M0 | G2 | MSS | KRAS | n.a. / n.a. | Rostock |
| HROC103 | Tu / N | 44 | M | Caucasian | Rectum | T2N1M0 | G2 | MSS | TP53, APC | 8.4 / 8.7 | Rostock |
| HROC105 | Tu / N | 52 | M | Caucasian | Rectum | T4N2M1 | G2 | MSS | wt | n.a. / n.a. | Rostock |
| HROC106 | Tu / N | 78 | M | Caucasian | Sigmoid | T3N2M1 | G2 | MSS | KRAS | 9.2 / 8.9 | Rostock |
| HROC107 | Tu / N | 74 | M | Caucasian | Sigmoid | T3N2M1 | G2 | MSS | KRAS, TP53, APC | 5.8 / 8.3 | Rostock |
| HROC108 | Tu / N | 81 | F | Caucasian | Ascending | T3N0M0 | G3 | MSI | wt | n.a. / 9.2 | Rostock |
| HROC110 | Tu / N | 88 | F | Caucasian | Ascending | T3N0M0 | G2 | MSI | BRAF | 5.4 / 7.4 | Rostock |
| HROC112 | Tu / N | 78 | F | Caucasian | Coecum | T3N2M1 | G2 | MSS | wt | 7.5 / 6.5 | Rostock |
| HROC125 | Tu / N | 84 | F | Caucasian | Recto-sigmoid | T3N1M0 | G2 | MSS | wt | 8.6 / 8.5 | Rostock |
| HROC126 | Tu / N | 58 | F | Caucasian | Rectum | T3N1M0 | G2 | MSS | wt | 8.9 / 8.8 | Rostock |
| HROC129 | Tu / N | 76 | F | Caucasian | Transverse | T3N1M0 | G2 | MSS | wt | 8.7 / 9.7 | Rostock |
| HROC130 | Tu / N | 60 | M | Caucasian | Sigmoid | T3N1M1 | G3 | MSI | wt | 5.5 / n.a. | Rostock |
| HROC131 | Tu / N | 75 | F | Caucasian | Ascending | T3N1M0 | G3 | MSI | BRAF | 9.2 / 8.8 | Rostock |
| HROC135 | Tu / N | 73 | M | Caucasian | Ascending | T3N1M0 | G2 | MSS | KRAS | 8.6 / 8.7 | Rostock |
| HROC138 | Tu / N | 81 | M | Caucasian | Descending | T3N0 | G2 | MSS | wt | 9.7 / 8.9 | Rostock |
| HROC141 | Tu / N | 81 | M | Caucasian | Rectum | T3N2 | G2 | MSS | wt | 8.3 / 8.3 | Rostock |
| HROC143 | Tu / N | 74 | M | Caucasian | Rectum | T1N0M0 | G3 | MSS | KRAS | 8.4 / 9.4 | Rostock |
| HROC146 | Tu / N | 80 | M | Caucasian | Ascending | T3N0M0 | G3 | MSI | BRAF | 9.8 / 7.8 | Rostock |
| HROC147 | Tu / N | 54 | M | Caucasian | Recto-sigmoid | T3N2M1 | G3 | MSS | KRAS | n.a. / 3.8 | Rostock |
| HROC148 | Tu / N | 81 | F | Caucasian | Rectum | T2 | n.a. | MSS | KRAS | 7.6 / 7.6 | Rostock |
| HROC150 | Tu / N | 63 | M | Caucasian | Transverse | T4N2M1 | G2 | MSS | KRAS | n.a. / 10.0 | Rostock |
| HROC154 | Tu / N | 63 | F | Caucasian | Sigmoid | T4N0M0 | G2 | MSS | KRAS | n.a. / n.a. | Rostock |
| HROC162 | Tu / N | 82 | M | Caucasian | Hepatic flexure | T3N0M0 | G2 | MSS | wt | 9.3 / 9.0 | Rostock |
| HROC168 | Tu / N | 67 | M | Caucasian | Sigmoid | T3N1M1 | G2 | MSS | KRAS | 8.4 / 8.4 | Rostock |
| HROC169 | Tu / N | 85 | F | Caucasian | Ascending | T4N2M1 | G3 | MSS | KRAS | 9.6 / 9.0 | Rostock |
| HROC170 | Tu / N | 68 | F | Caucasian | Ascending | T3N0M0 | G3 | MSI | BRAF | 9.9 / 9.5 | Rostock |
| HROC172 | Tu / N | 83 | M | Caucasian | Recto-sigmoid | T3N0M1 | G2 | MSS | wt | 8.9 / 8.0 | Rostock |
| HROC173 | Tu / N | 45 | M | Caucasian | Ascending | T4N2M1 | G3 | MSS | KRAS | n.a. / 7.3 | Rostock |
| HROC175 | Tu / N | 87 | F | Caucasian | Coecum | T4N0M0 | G3 | MSI | BRAF | 7.2 / 7.3 | Rostock |
| HROC176 | Tu / N | 71 | M | Caucasian | Hepatic flexure | T3N2M0 | G2 | MSS | KRAS | 6.2 / 5.6 | Rostock |
| HROC178 | Tu / N | 58 | F | Caucasian | Coecum | T4N2 | G3 | MSS | wt | 8.0 / 6.7 | Rostock |
| HROC185 | Tu / N | 90 | F | Caucasian | Descending | T2N0M1 | G3 | MSS | wt | n.a. / 6.3 | Rostock |
| HROC186 | Tu / N | 85 | F | Caucasian | Rectum | T4N2M0 | G3 | n.a. | n.a. | 7.8 / 5.5 | Rostock |
| HROC199 | Tu / N | 80 | M | Caucasian | Sigmoid | T3N0 | G2 | MSS | wt | 7.5 / 5.1 | Rostock |
| HROC204 | Tu / N | 60 | M | Caucasian | Hepatic flexure | T3N1 | G3 | MSI | BRAF | 8.4 / 7.6 | Rostock |
| HROC206 | Tu / N | 83 | F | Caucasian | Sigmoid | T3N0M0 | G2 | MSS | wt | 9.1 / 7.1 | Rostock |
| HROC208 | Tu / N | 59 | M | Caucasian | Sigmoid | T2N0 | G2 | MSS | KRAS | 6.7 / 7.1 | Rostock |
| HROC210 | Tu / N | 61 | M | Caucasian | Recto-sigmoid | T3N0M0 | G2 | MSS | wt | 7.9 / n.a. | Rostock |
| HROC212 | Tu / N | 74 | F | Caucasian | Coecum | T4N2M1 | G3 | MSI | BRAF | n.a. / 5.9 | Rostock |
| HROC213 | Tu / N | 82 | F | Caucasian | Coecum | T4N0M0 | G2 | MSS | wt | 7.2 / 7.8 | Rostock |
| HROC217 | Tu / N | 73 | M | Caucasian | Ascending | T3N1M0 | G2 | MSS | wt | 8.4 / 8.5 | Rostock |
| HROC221 | Tu / N | 64 | F | Caucasian | Ascending | T2N0 | G2 | MSI | n.a. | 6.7 / 6.1 | Rostock |
| HROC223 | Tu / N | 58 | M | Caucasian | Rectum | T2N0 | G2 | MSS | wt | 4.9 / 8.9 | Rostock |
| HROC224 | Tu / N | 77 | M | Caucasian | Rectum | T3N2M1 | G2 | MSS | KRAS | 8.2 / 8.6 | Rostock |
| HROC225 | Tu / N | 76 | F | Caucasian | Rectum | T4N2M1 | G3 | MSS | wt | 9.6 / 7.2 | Rostock |
| HROC228 | Tu / N | 54 | M | Caucasian | Sigmoid | T3N0 | G2 | MSS | wt | 7.4 / 8.0 | Rostock |
| HROC231 | Tu / N | 75 | M | Caucasian | Rectum | n.a. | n.a. | n.a. | n.a. | 7.0 / 3.9 | Rostock |
| HROC232 | Tu / N | 79 | M | Caucasian | Coecum | T4N1M0 | G2 | MSS | n.a. | 5.8 / 6.3 | Rostock |
| HROC236 | Tu / N | 75 | F | Caucasian | Ascending | T3N0M0 | G3 | MSI | n.a. | 8.0 / 8.4 | Rostock |
| HROC239 | Tu / N | 72 | F | Caucasian | Rectum | T4N2M0 | G2 | MSS | n.a. | 8.7 / 9.0 | Rostock |
| HROC245 | Tu / N | 73 | M | Caucasian | Rectum | T3N0M0 | G3 | MSS | n.a. | 7.6 / 8.0 | Rostock |
| HROC248 | Tu / N | 65 | M | Caucasian | Coecum | T4N2M1 | G2 | MSS | n.a. | 6.9 / 8.4 | Rostock |
| HROC250 | Tu / N | 64 | F | Caucasian | Sigmoid | T3N0M0 | G2 | n.a. | n.a. | 8.2 / 7.8 | Rostock |
| HROC251 | Tu / N | 85 | M | Caucasian | Ascending | T3N0M0 | G1 | MSI | n.a. | 9.1 / 7.0 | Rostock |
| HROC252 | Tu1/N | 45 | M | Caucasian | Descending | T4N0M0 | G3 | MSI | n.a. | 7.4 / 7.4 | Rostock |
| HROC252 | Tu2/N | 45 | M | Caucasian | Sigmoid | T4N0M0 | G3 | MSI | n.a. | 8.8 / 7.4 | Rostock |
| HROC252 | Tu3/N | 45 | M | Caucasian | Rectum | T4N0M0 | G3 | MSI | n.a. | 8.6 / 7.4 | Rostock |
| HROC253 | Tu / N | 61 | M | Caucasian | Rectum | T4N1M1 | G3 | MSS | n.a. | 4.7 / 3.8 | Rostock |
| HROC256 | Tu / N | 70 | M | Caucasian | Descending | T3N0M0 | G2 | MSI | n.a. | 8.9 / 8.0 | Rostock |
| HROC257 | Tu / N | 84 | F | Caucasian | Ascending | T4N2 | G3 | MSI | n.a. | 8.1 / 8.5 | Rostock |
| HROC258 | Tu / N | 77 | F | Caucasian | Recto-sigmoid | T3N0 | G2 | MSS | n.a. | 7.8 / 7.6 | Rostock |
| HROC259 | Tu / N | 75 | M | Caucasian | Sigmoid | T3N2 | G2 | MSS | n.a. | 7.6 / 5.3 | Rostock |
| HROC260 | Tu / N | 79 | F | Caucasian | Ascending | T3N0M0 | G2 | MSS | n.a. | 9.0 / 8.3 | Rostock |
| HROC264 | Tu / N | 71 | F | Caucasian | Hepatic flexure | T3N0M0 | G1 | MSS | n.a. | 7.5 / 7.2 | Rostock |
| HROC266 | Tu / N | 75 | M | Caucasian | Left flexure | T4N2M1 | G3 | MSS | n.a. | 6.0 / 7.5 | Rostock |
| HROC267 | Tu / N | 85 | M | Caucasian | Transverse | T3N0M0 | G2 | MSS | n.a. | 7.5 / 6.8 | Rostock |
| HROC268 | Tu / N | 89 | F | Caucasian | Rectum | T3N0M0 | G2 | MSS | n.a. | 7.7 / 7.4 | Rostock |
| HROC269 | Tu / N | 76 | F | Caucasian | Hepatic flexure | T3N1M0 | G2 | MSI | n.a. | 8.7 / 3.4 | Rostock |
| HROC271 | Tu / N | 79 | F | Caucasian | Ascending | T2N0M0 | G2 | MSS | n.a. | 6.7 / 6.3 | Rostock |
| HROC272 | Tu / N | 73 | M | Caucasian | Ascending | T3N1M0 | G2 | MSS | n.a. | n.a. / 2.6 | Rostock |
| HROC277 | Tu / N | 77 | M | Caucasian | Coecum | T4N0M1 | G2 | MSS | n.a. | 9.3 / 8.7 | Rostock |
| HROC278 | Tu / N | 76 | F | Caucasian | Ascending | T4N2M1 | G3 | MSS | n.a. | 8.7 / 8.9 | Rostock |
| HROC279 | Tu / N | 90 | F | Caucasian | Sigmoid | T4N0M0 | G3 | MSI | n.a. | 8.9 / 8.9 | Rostock |
| HROC281 | Tu / N | 77 | M | Caucasian | Colon | T2N0 | G2 | MSS | n.a. | 8.8 / 7.6 | Rostock |
| HROC282 | Tu / N | 84 | F | Caucasian | Rectum | n.a. | n.a. | MSS | n.a. | 8.8 / 8.7 | Rostock |
| HROC283 | Tu / N | 48 | F | Caucasian | Sigmoid | T3N1M0 | G2 | MSS | n.a. | 8.1 / 9.0 | Rostock |
| HROC285 | Tu / N | 30 | F | Caucasian | Colon | T4N2M1 | G2 | n.a. | n.a. | 8.6 / 9.2 | Rostock |
| HROC286 | Tu / N | 82 | M | Caucasian | Rectum | T3N0M0 | G2 | MSS | n.a. | 8.1 / 8.5 | Rostock |
| HROC287 | Tu / N | 75 | M | Caucasian | Rectum | T3N2M0 | G2 | MSS | n.a. | 8.5 / 4.0 | Rostock |
| HROC288 | Tu / N | 84 | F | Caucasian | Sigmoid | T3N1M0 | G3 | MSS | n.a. | 8.3 / 6.6 | Rostock |
| 1 | A/N | 26 | F | Caucasian | n.a. | Adenoma | n.a. | n.a. | n.a. | 6.7 / 4.6 | Lyon |
| 3 | A/N | 54 | F | Caucasian | n.a. | Adenoma | n.a. | n.a. | n.a. | 2.2 / 2.1 | Lyon |
| 5 | A/N | 41 | M | Caucasian | n.a. | Adenoma | n.a. | n.a. | n.a. | 4.1 / 2.3 | Lyon |
| 8 | A/N | 32 | M | Caucasian | n.a. | Adenoma | n.a. | n.a. | n.a. | 3.7 / 4.1 | Lyon |
| 9 | A/N | 62 | M | Caucasian | n.a. | Adenoma | n.a. | n.a. | n.a. | 3.9 / 2.1 | Lyon |
| 12 | A/N | 46 | M | Caucasian | n.a. | Adenoma | n.a. | n.a. | n.a. | 3.0 / 2.7 | Lyon |
| 13 | A/N | 87 | M | Caucasian | n.a. | Adenoma | n.a. | n.a. | n.a. | 6.2 / 1.9 | Lyon |
| 15 | A/N | 81 | F | Caucasian | n.a. | Adenoma | n.a. | n.a. | n.a. | 2.7 / 2.4 | Lyon |
| 16 | A/N | 63 | M | Caucasian | n.a. | Adenoma | n.a. | n.a. | n.a. | 4.0 / 6.4 | Lyon |
| 17 | A/N | 63 | M | Caucasian | n.a. | Adenoma | n.a. | n.a. | n.a. | 8.4 / 5.2 | Lyon |
| 18 | A/N | 40 | F | Caucasian | n.a. | Adenoma | n.a. | n.a. | n.a. | n.a. / 2.7 | Lyon |
| 19 | A/N | 53 | M | Caucasian | n.a. | Adenoma | n.a. | n.a. | n.a. | 7.1 / 3.9 | Lyon |
| HROC120 | A/N | 70 | M | Caucasian | Left flexure | Adenoma | n.a. | n.a. | n.a. | 8.8 / 7.6 | Rostock |
| HROC124 | A/N | 79 | F | Caucasian | Rectum | Adenoma | n.a. | n.a. | n.a. | 8.9 / 8.6 | Rostock |
| HROC163 | A/N | 84 | M | Caucasian | Rectum | Adenoma | n.a. | n.a. | n.a. | 9.2 / 8.6 | Rostock |
| HROC191 | A | 67 | M | Caucasian | Recto-sigmoid | Adenoma | n.a. | n.a. | n.a. | 8.4 | Rostock |
| HROC196 | A/N | 74 | M | Caucasian | Coecum | Adenoma | n.a. | n.a. | n.a. | n.a. / 7.4 | Rostock |
| HROC209 | A/N | 76 | M | Caucasian | Coecum | Adenoma | n.a. | n.a. | n.a. | 8.9 / 7.8 | Rostock |
| HROC225 | A | 76 | F | Caucasian | Rectum | Adenoma | n.a. | n.a. | n.a. | 8.7 | Rostock |
| HROC248 | A | 65 | M | Caucasian | Ascending | Adenoma | n.a. | n.a. | n.a. | 8.7 | Rostock |
| HROC263 | A/N | 52 | M | Caucasian | Recto-sigmoid | Adenoma | n.a. | n.a. | n.a. | 9.4 / 9.1 | Rostock |
| HROC72 | Tu/Met | 61 | M | Caucasian | Liver | Metastasis | n.a. | n.a. | n.a. | 9.3 / 9.4 | Rostock |
| HROC112 | Met/N | 79 | F | Caucasian | Liver | Metastasis | n.a. | n.a. | n.a. | 9.6 / 8.1 | Rostock |
| HROC130 | Tu/Met | 61 | M | Caucasian | Liver | Metastasis | n.a. | n.a. | n.a. | 5.5 / 7.2 | Rostock |
| HROC147 | Tu/Met | 42 | M | Caucasian | Liver | Metastasis | n.a. | n.a. | n.a. | n.a. / 8.2 | Rostock |
| HROC157 | Met/N | 42 | M | Caucasian | Liver | Metastasis | n.a. | n.a. | n.a. | 8.7 / 8.6 | Rostock |
| HROC177 | Met/N | 63 | F | Caucasian | Liver | Metastasis | n.a. | n.a. | n.a. | 7.7 / 7.4 | Rostock |
| HROC203 | Met/N | 70 | M | Caucasian | Liver | Metastasis | n.a. | n.a. | n.a. | 7.4 / 7.7 | Rostock |
| HROC215 | Met/N | 28 | F | Caucasian | Liver | Metastasis | n.a. | n.a. | n.a. | 7.1 / 6.5 | Rostock |
| HROC224 | Tu/Met | 77 | M | Caucasian | Liver | Metastasis | n.a. | n.a. | n.a. | 7.4 / 8.2 | Rostock |
| HROC230 | Met R1/N | 74 | M | Caucasian | Liver | Metastasis | n.a. | n.a. | n.a. | 8.4 / 6.7 | Rostock |
| HROC230 | Met R2/N | 74 | M | Caucasian | Liver | Metastasis | n.a. | n.a. | n.a. | 9.1 / 8.1 | Rostock |
| HROC253 | Met/N | 61 | M | Caucasian | Liver | Metastasis | n.a. | n.a. | n.a. | 9.1 / 3.8 | Rostock |
| HROC277 | Tu/Met | 77 | M | Caucasian | Liver | Metastasis | n.a. | n.a. | n.a. | 9.3 / 8.1 | Rostock |
| HROC284 | Met/N | 67 | F | Caucasian | Liver | Metastasis | n.a. | n.a. | n.a. | 8.2 / n.a. | Rostock |
| HROC301 | Met | 58 | M | Caucasian | Liver | Metastasis | n.a. | n.a. | n.a. | 8.2 | Rostock |
| HROC304 | Met | 71 | F | Caucasian | Liver | Metastasis | n.a. | n.a. | n.a. | 8.4 | Rostock |
| HROGas01 | Tu / N | 71 | M | Caucasian | Stomach | Tumor | n.a. | n.a. | n.a. | 8.5 / 10.0 | Rostock |
| HROGas03 | Tu / N | 80 | F | Caucasian | Stomach | Tumor | n.a. | n.a. | n.a. | 9.1 / 9.9 | Rostock |
| HROGas04 | Tu / N | 71 | M | Caucasian | Stomach | Tumor | n.a. | n.a. | n.a. | 10.0 / 9.7 | Rostock |
| HROHep01 | Tu / N | 74 | M | Caucasian | Liver | Tumor | n.a. | n.a. | n.a. | 9.5 / 9.5 | Rostock |
| HROHep01 R1 | Tu / N | 75 | M | Caucasian | Liver | Tumor | n.a. | n.a. | n.a. | 9.8 / 9.5 | Rostock |
| HROHep03 | Tu / N | 71 | F | Caucasian | Liver | Tumor | n.a. | n.a. | n.a. | 9.6 / 8.4 | Rostock |
| HROHep06 | Tu / N | 62 | M | Caucasian | Liver | Tumor | n.a. | n.a. | n.a. | 9.0 / 8.0 | Rostock |
| HROLu02 | Tu | 72 | F | Caucasian | Lung | Tumor | n.a. | n.a. | n.a. | 8.5 | Rostock |
| HROLu04 | Tu / N | 74 | F | Caucasian | Lung | Tumor | n.a. | n.a. | n.a. | 10.0 / 8.0 | Rostock |
| HROLu05 | Tu / N | 71 | F | Caucasian | Lung | Tumor | n.a. | n.a. | n.a. | 9.6 / 7.4 | Rostock |
| HROLu06 | Tu / N | 75 | M | Caucasian | Lung | Tumor | n.a. | n.a. | n.a. | 10.0 / n.a. | Rostock |
| HROLu07 | Tu / N | 57 | M | Caucasian | Lung | Tumor | n.a. | n.a. | n.a. | 7.9 / 7.9 | Rostock |
| HROP17 | Tu / N | 75 | F | Caucasian | Pancreas | Tumor | n.a. | n.a. | n.a. | 7.3 / 7.7 | Rostock |
| HROP27 | Tu / N | 70 | F | Caucasian | Pancreas | Tumor | n.a. | n.a. | n.a. | 7.0 / 4.3 | Rostock |
| HROP41 | Tu / N | 73 | F | Caucasian | Pancreas | Tumor | n.a. | n.a. | n.a. | 8.3 / 7.0 | Rostock |
